# Fragment screening for a protein-protein interaction inhibitor to WDR5

**DOI:** 10.1063/1.5122849

**Published:** 2019-11-14

**Authors:** Matthew L. Dennis, Benjamin J. Morrow, Olan Dolezal, Anthony N. Cuzzupe, Alexandra E. Stupple, Janet Newman, John Bentley, Meghan Hattarki, Stewart D. Nuttall, Richard C. Foitzik, Ian P. Street, Paul A. Stupple, Brendon J. Monahan, Thomas. S. Peat

**Affiliations:** 1 Commonwealth Scientific and Industrial Research Organisation (CSIRO), Biomedical Program, Parkville, Victoria 3052, Australia; 2 Medicinal Chemistry Theme, Monash Institute of Pharmaceutical Sciences, Monash University, Parkville, Victoria 3052, Australia; 3 Cancer Therapeutics CRC, Parkville, Victoria 3052, Australia; 4 SYNthesis med chem (Australia) Pty Ltd, Bio21 Institute, 30 Flemington Road, Parkville, Victoria 3052, Australia; 5 The Walter and Eliza Hall Institute of Medical Research, Parkville, Melbourne, Victoria 3052, Australia; 6 Department of Medical Biology, University of Melbourne, Parkville, Victoria 3052, Australia

## Abstract

The WD40-repeat protein WDR5 scaffolds various epigenetic writers and is a critical component of the mammalian SET/MLL histone methyltransferase complex. Dysregulation of the MLL1 catalytic function is associated with mixed-lineage leukemia, and antagonism of the WDR5-MLL1 interaction by small molecules has been proposed as a therapeutic strategy for MLL-rearranged cancers. Small molecule binders of the “WIN” site of WDR5 that cause displacement from chromatin have been additionally implicated to be of broader use in cancer treatment. In this study, a fragment screen with Surface Plasmon Resonance (SPR) was used to identify a highly ligand-efficient imidazole-containing compound that is bound in the WIN site. The subsequent medicinal chemistry campaign—guided by a suite of high-resolution cocrystal structures with WDR5—progressed the initial hit to a low micromolar binder. One outcome from this study is a moiety that substitutes well for the side chain of arginine; a tripeptide containing one such substitution was resolved in a high resolution structure (1.5 Å) with a binding mode analogous to the native tripeptide. SPR furthermore indicates a similar residence time (*k*_d_ = ∼0.06 s^−1^) for these two analogs. This novel scaffold therefore represents a possible means to overcome the potential permeability issues of WDR5 ligands that possess highly basic groups like guanidine. The series reported here furthers the understanding of the WDR5 WIN site and functions as a starting point for the development of more potent WDR5 inhibitors that may serve as cancer therapeutics.

## I. INTRODUCTION

A “cellular multitasker” with many binding partners, the scaffolding protein WDR5 is best studied in the context of histone methylation as part of the SET1/MLL (mixed-lineage leukemia) complexes.^1^ Each complex has a unique SET/MLL subunit and binds a consistent set of WRAD proteins (WDR5, RbBP5, Ash2L, and DPY30), which enables the methylation of lysine 4 of Histone 3 (H3K4me).^2–4^ This histone modification is highly enriched at active promoters near transcription start sites (TSSs) and positively correlated with transcription.^5^ WDR5 is key in this process, particularly for MLL1; the absence of WDR5 abolishes the structural integrity and activity of the MLL1 complex.^2,4^ Dysregulation of MLL1 is associated with acute myeloid and lymphoblastic leukemias, with MLL-rearranged (MLL-r) cancers retaining a wildtype copy of MLL1 whose activity has separately been ascertained as essential^6,7^ or nonessential^8^ for leukemogenesis.

WDR5 comprises a 7-bladed β-propeller fold, each blade composed of a four-stranded antiparallel β-sheet (the WD40 motif).^9–11^ Cavities on each face, namely, the “WDR5-binding motif” (WBM) and “WDR5-interacting” (WIN) sites, are the interfaces key to its scaffolding role.^12,13^ In the context of the MLL1 complex, these sites bind RbBP5 and the MLL1 subunit, respectively.^13,14^ Although it is hypothesized that there are proteins that interact with the “sides” of WDR5,^1^ structurally interrogated binding partners to date relate only to the WBM and WIN sites. One or both of these sites are appropriate for the scaffolding of various complexes, including the NSL (nonspecific lethal),^15^ NuRD (nucleosome remodeling and deacetylase),^16^ and ATAC (Ada2a-containing) complexes.^17^ WDR5 is also known to bind MYC, an overexpressed oncogene transcription factor, via the WBM site; mutations of this interaction have been demonstrated to prevent MYC from associating with target genes.^18^ WBM-site inhibitors therefore have potential as anticancer agents—being able to directly disrupt the interaction of c-MYC to WDR5 and thus impede the MYC function in cancer cells.^18,19^ However, WDR5 inhibitors to date have consisted only of WIN-site binders. The WIN site is dominated by a conserved arginine pocket, where the guanidine headgroup sits in a “phenylalanine clamp”^11^ composed of Phe133 and Phe263. The “WIN-motif” has some flexibility regarding arginine-adjacent residues, but the arginine itself is critical; studies of peptide binding to WDR5 show a dramatic loss of affinity if the arginine is mutated.^20,21^ The first WDR5 inhibitors developed were accordingly peptides and peptidomimetics that built from this arginine-critical WIN-motif, guided by the co-crystal structures of WDR5 with MLL1 and H3 peptides. One such peptidomimetic, MM-401, was shown to be selective against MLL-r cancer cells and cell lines *in vitro*.^22^ Small molecules that bind the WIN site have focused on a series of *N*-methyl piperazines identified from a fluorescence polarization assay screen^23^ that led to OICR-9429.^24–27^ This compound inhibits the histone methyltransferase (HMT) activity of MLL1 *in vitro* and reduces the viability of primary human AML cells bearing C/EBPα mutations^24^ and inhibits “gain-of-function” p53 cell growth.^26^ Recently, an NMR-based fragment screen identified several high-affinity WIN-site binders.^28,29^ These compounds show cellular activity through displacement of WDR5 from chromatin, an effect that gives WDR5 inhibitors therapeutic relevance beyond MLL-r cancers.^29^ These studies have validated WDR5—in particular, the WIN site—as a promising anticancer target and advocate further research of novel inhibitors.^30^ The present study used a fragment screening approach with SPR (Surface Plasmon Resonance). Of the hits from this screen, one compound [(2-butyl-1*H*-imidazol-5-yl)methanol] was chosen as the most promising to advance. Cocrystal structures of the initial hit and subsequent derivatives enabled structure-guided optimization to **37** (*K*_D_ = 4.5 *μ*M), which represents a useful starting point for the development of clinically relevant WDR5 inhibitors. The structure-activity relationship (SAR) of this series in the context of the structural data is discussed. The imidazole-containing headgroup of this series was investigated as a replacement for arginine; one such imidazole-peptide hybrid was cocrystallized with WDR5 and found to maintain the binding mode of analogous peptides. This novel moiety thus potentially has additional utility as a general tool to overcome the poor permeability associated with highly basic groups like arginine.

## II. RESULTS AND DISCUSSION

**FIG. 1.**
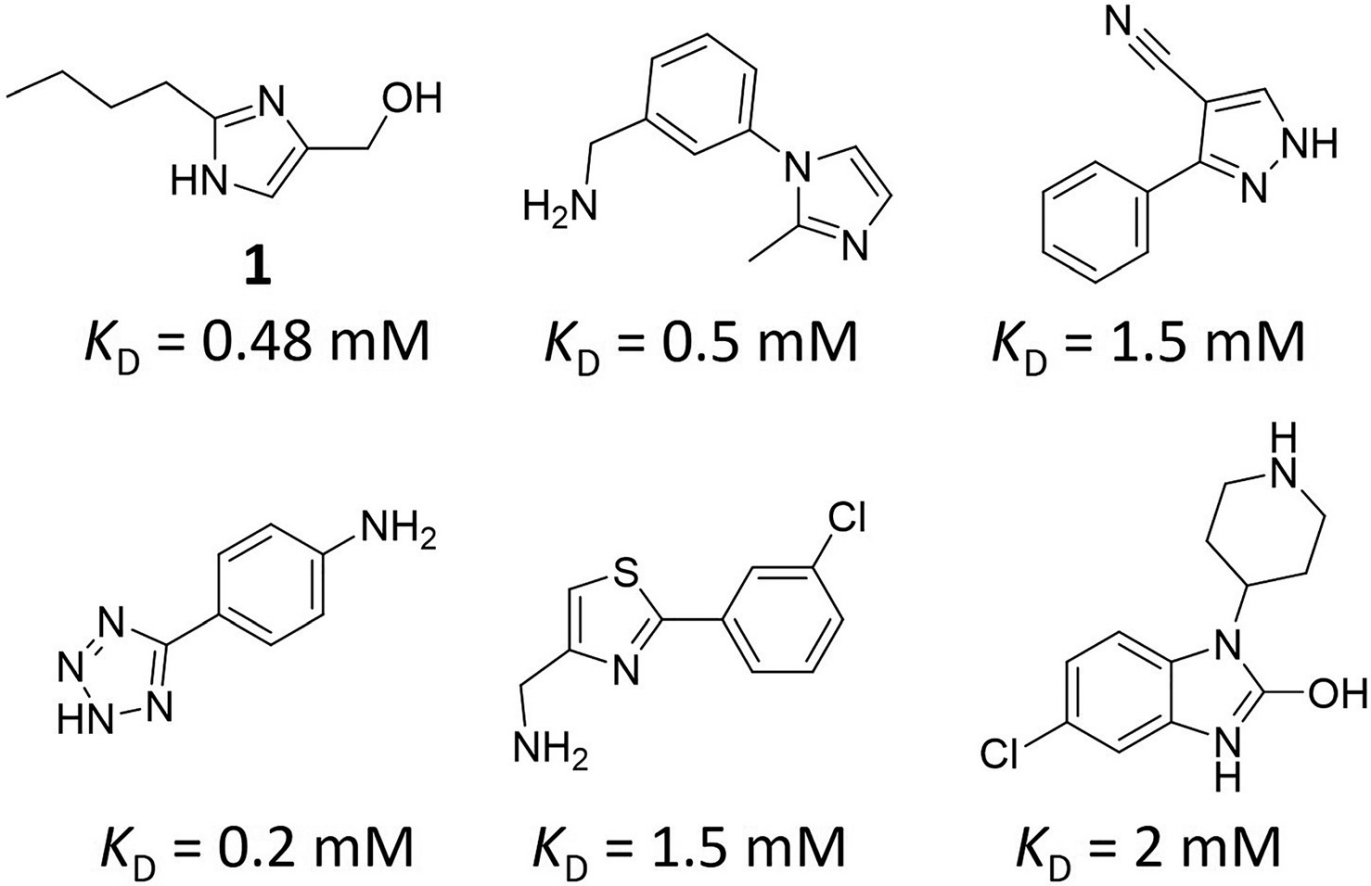
Chemical structures of selected hit compounds from the initial SPR screen.

**FIG. 2.**
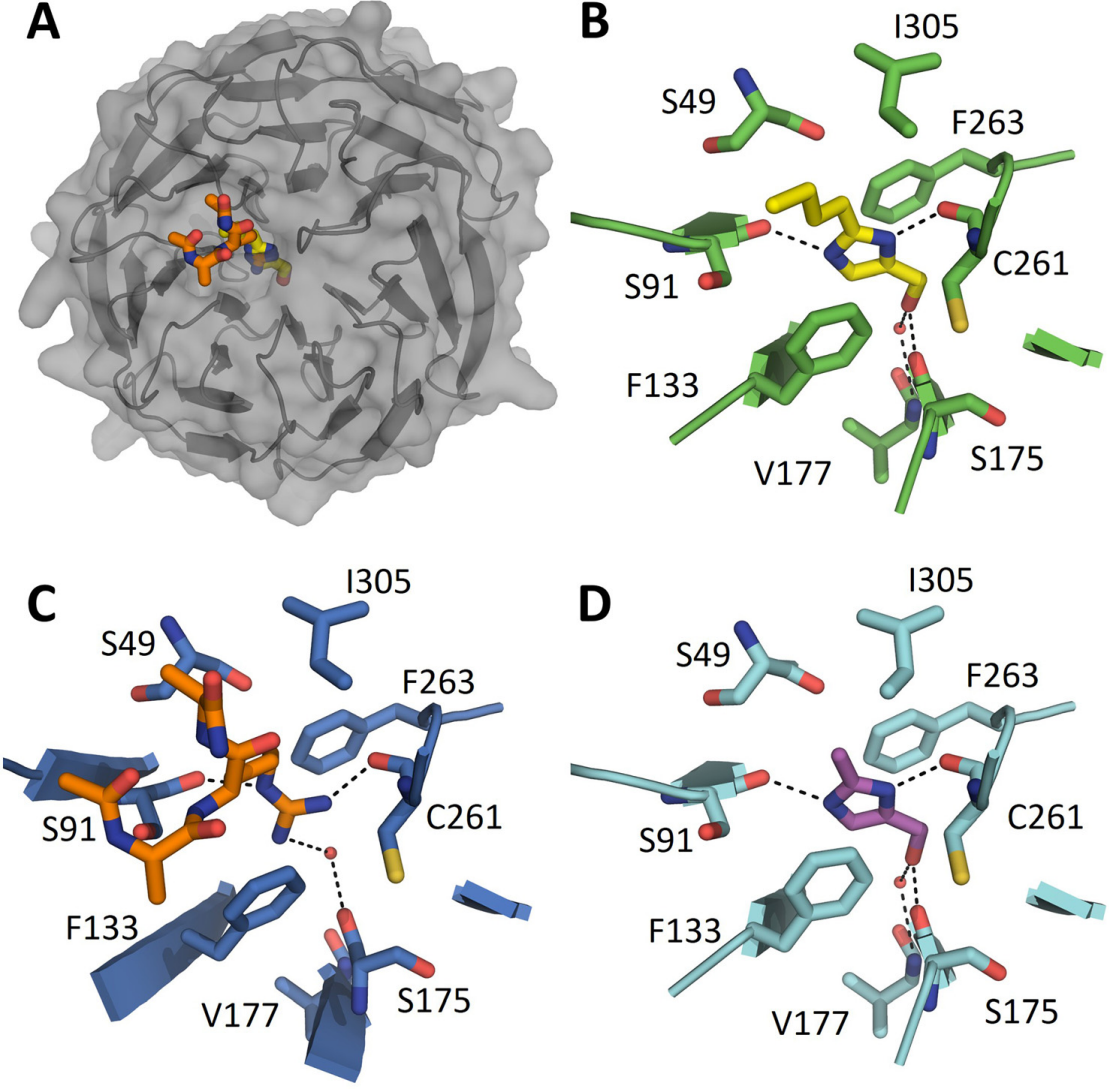
(a) Overlay of WDR5 cocrystal structures bound to the MLL peptide (3EG6,^14^ Ac-ARA-NH_2_ displayed), **1**, and **2**. Binding mode of (b) **1**, (c) the MLL1 peptide, and (d) **2**.

Fragment screening of WDR5 was performed using SPR, with compounds tested at a single concentration and potential hits re-tested in a dosage format. Screening focused on the Maybridge Ro3 and CSIRO fragment libraries,^31^ a total of 2256 compounds, with 12 of these identified as hits (0.53% hit rate, Fig. 1). Compound **1** presented a high ligand efficiency (*K*_D_ = 480 *μ*M, LE = 0.41 kcal·mol^−1^·heavy atom^−1^) and—following the confirmation of binding through cocrystallization with WDR5 (Table I)—was selected as the most promising hit to advance. Compound **1** was determined to bind in the WIN site, the imidazole ring stacking between F133 and F263 (the “phenylalanine clamp”^11^), thus occupying a similar space to the key arginine of the WIN motif (Fig. 2). The nitrogen atoms of the imidazole mimic the arginine headgroup, donating hydrogen bonds to the backbone of S91 and C261, while the hydroxyl moiety projects deeper into the pocket, hydrogen bonding directly to S175 and via a water molecule to V177. The hydroxyl displaces a water observed in the structures of WDR5 bound to MLL1 and H3K4 peptides.^10,14,32^ The butyl tail is oriented out of the pocket, thereby picking up additional hydrophobic interactions to S49, F133, and I305. The protein in the WDR5/**1** complex displayed strong electron density from residue K32. A truncated WDR5 construct (32–334) was therefore expressed and used in crystallization. The majority of the structures described in this report are from this truncated construct, which routinely diffracted to high resolution (Table I and supplementary Table S1).

**TABLE I.** Summary of crystallographic data for WDR5 cocrystal structures.

Compound	Resolution (Å)	R_work_/R_free_	PDB accession code
**1**	2.04	17.3/20.6	6PG3
**2**	1.60	14.7/17.6	6PG4
**16**	1.67	18.7/20.7	6PG8
**17**	2.45	19.6/24.3	6PG7
**24**	1.76	18.2/21.3	6PGE
**29**	1.68	23.3/26.9	6PG6
**30**	1.75	17.4/21.7	6PG9
**31**	1.73	17.4/21.7	6PGB
**32**	2.45	19.7/25.0	6PGA
**35**	1.99	15.6/20.2	6PG5
**37**	1.81	20.3/24.8	6PGC
**49**	1.54	14.8/16.9	6PGF
**55**	1.50	15.1/16.8	6PGD

To probe the binding requirements of **1**, prior to exploring elaborated derivatives, various fragment analogs were tested (Table II). Trimming the butyl tail of **1** to a methyl did not impair binding (**2,** *K*_D_ = 260 *μ*M), in contrast to the des-methyl (**3**, *K*_D_ > 3 mM), indicating the (2-methyl-imidazol-4-yl)methanol core as a highly ligand efficient starting point (LE = 0.61). Various imidazoles and other heterocycles, as well as isosteres of the hydroxyl moiety, displayed a general loss of affinity. This suggested rigid requirements for the hydrogen bonding network, at least for this series' binding mode; changing the nitrogen pattern to remove the hydrogen bond at either C261 or S91 (**4** and **5**, respectively) greatly reduced affinity. Removing the methanol moiety entirely leaving 2-methyl imidazole also reduced binding (**6**, *K*_D_ = 2.1 mM); however, this aligns with the reduced size of this compound and the high ligand efficiency of **2** is largely maintained (LE = 0.60). A cocrystal structure of **2** aligned well with **1** (ligand RMSD 0.2 Å) and placed the two solvent exposed positions—the 2-methyl and the hydroxyl groups—as the frontrunners for elaboration in the WDR5 channel.

**TABLE II.** SAR of the fragment core.

Compound	Chemical structure	KD (*μ*M)^a^
**1**	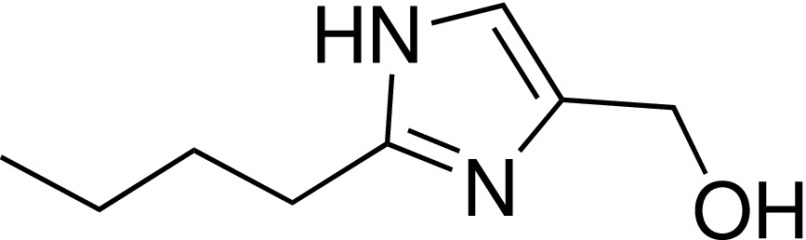	480 ± 30
**2**	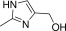	260 ± 20
**3**	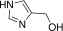	>3000^b^
**4**	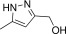	>3000
**5**	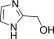	>3000
**6**	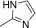	2100^c^

^a.^ Mean ± SE.

^b^ Estimated from single concentration (100 *μ*M) screen.

^c^ SE not reported (n = 1).

### A. Derivatization of the hydroxymethyl substituent of 2

**SCHEME 1.**
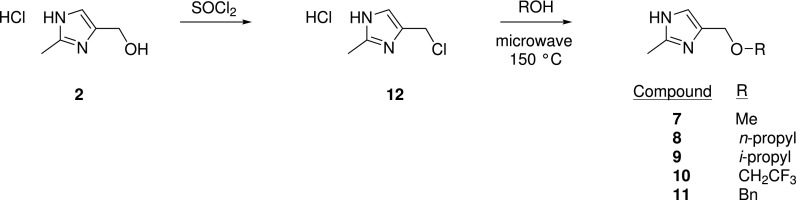
Synthesis of ether derivatives **7**–**11**.

From the crystal structures of **1** and **2**, it appeared possible to extend from the hydroxyl moiety, deeper into the WDR5 channel, with an eye to potentially form additional interactions with the hydrophobic residues F263, F219, or A176. Five analogs of **2** with hydrophobic substituents were tested to probe this vector (Table III and Scheme 1). Methyl, n-propyl, i-propyl, and 2,2,2,-trifluoroethyl groups all abrogated binding (**7**–**10**, *K*_D_ > 3 mM); however, a benzyl moiety (**11**, *K*_D_ = 950 *μ*M) was better tolerated. No cocrystal structure was obtained for **11**, and so a change in the binding orientation of the ligand cannot be ruled out. Further analogs would thus be required to convincingly demonstrate that extension from this vector could be tolerated with the appropriate functional group.

**TABLE III.** SAR of ether-derivatized 2.

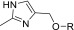		
Compound	R	*K*_D_ (*μ*M)^a^
**2**	H	260 ± 20
**7**	CH_3_	> 3000
**8**	n-propyl	> 3000
**9**	i-propyl	> 3000
**10**	CH_2_CF_3_	> 3000
**11**	Bn	950^b^

^a^ mean ± SE.

^b^ SE not reported (n = 1).

**TABLE IV.** SAR of derivatives of 2.

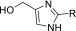		
Compound	R	*K*_D_ (*μ*M)^a^
**13**	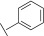	> 3000
**14**	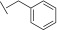	530^b^
**15**	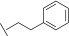	300 ± 40
**16**	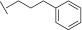	140 ± 30
**17**	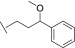	22 ± 6
**23**	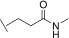	890^b^
**24**	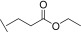	240 ± 10
**28**	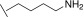	350 ± 40
**29**	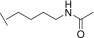	200 ± 10
**30**	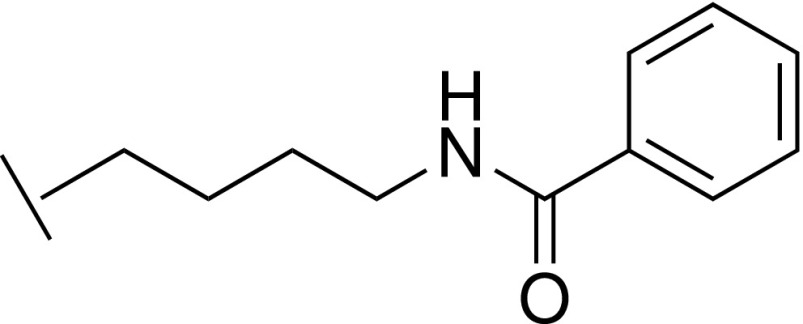	12 ± 1
**31**	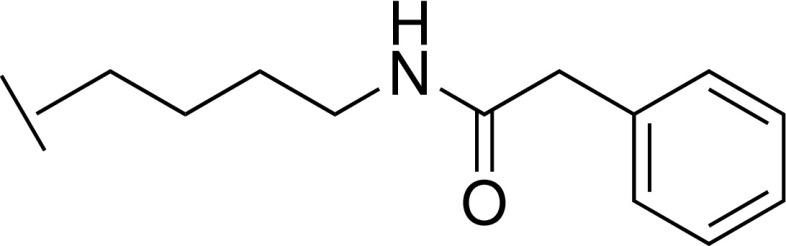	25 ± 3
**32**	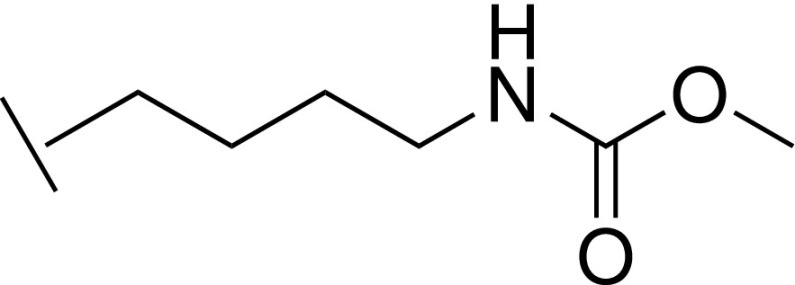	28.9 ± 0.5
**35**	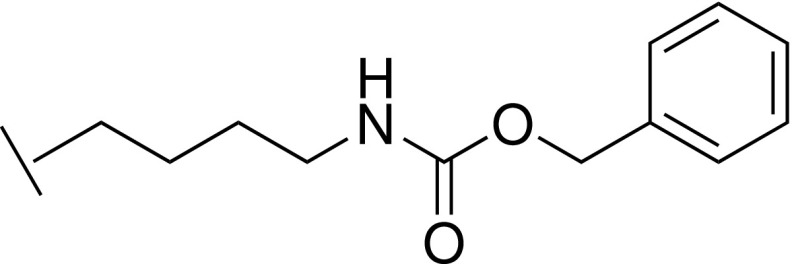	11 ± 1
**37**	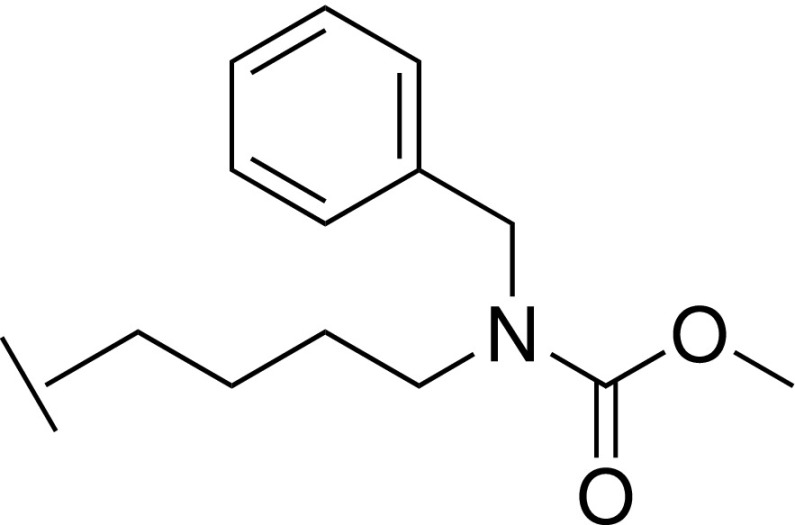	4.5 ± 0.3
**40**	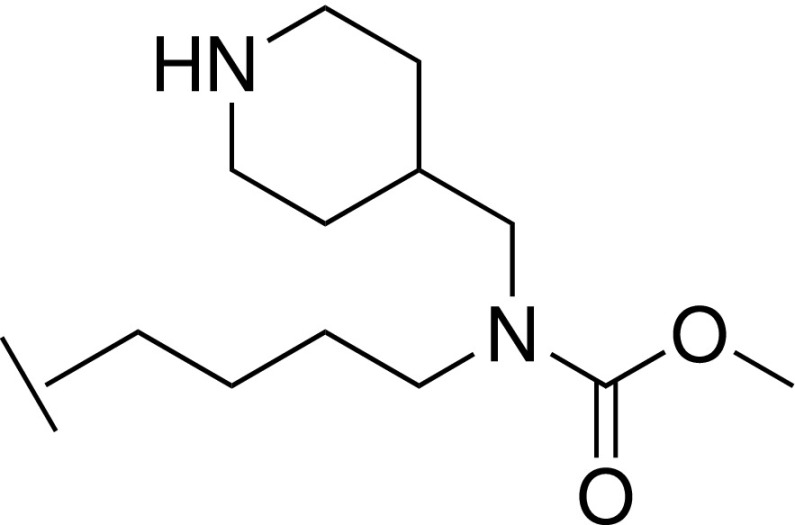	19 ± 3
**45**	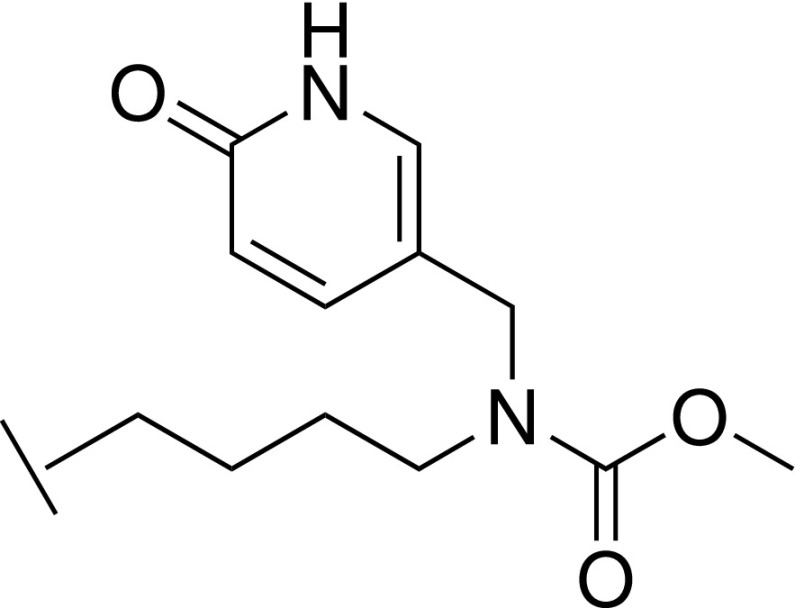	98.6 ± 0.6
**47**	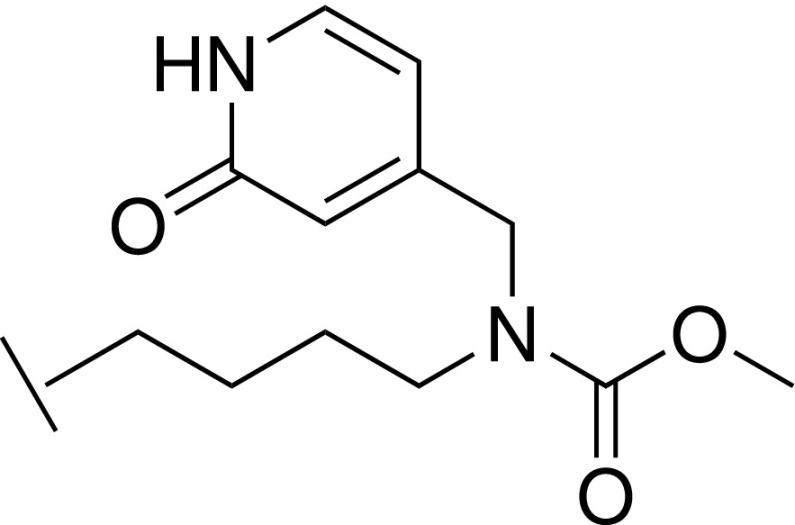	24.2 ± 0.2

^a^ mean ± SE.

^b^ SE not reported (n = 1).

### B. Aromatic substituents in the 2-position of 4(5)–(hydroxymethyl)imidazole

**SCHEME 2.**
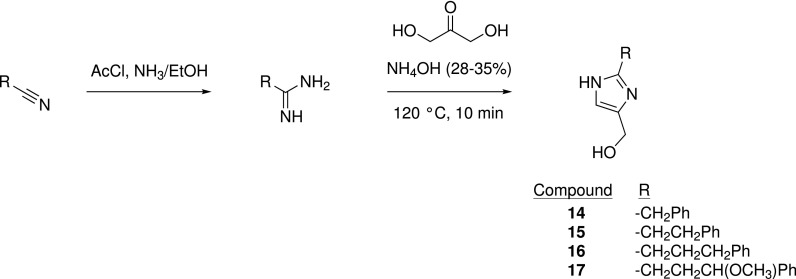
Synthesis of aryl derivatives **14**–**17**.

Synthetic efforts subsequently focused on extending from the 2-position of the imidazole ring to fill the WIN site. Considering that the alkyl chain of **1** contributed little to binding, initial efforts were directed at assessing whether an aromatic ring was tolerated at varying distances (0–3 atoms) from the imidazole fragment (Table IV). Condensation of dihydroxyacetone with variously substituted amidines afforded access to the final compounds (Scheme 2).

**FIG. 3.**
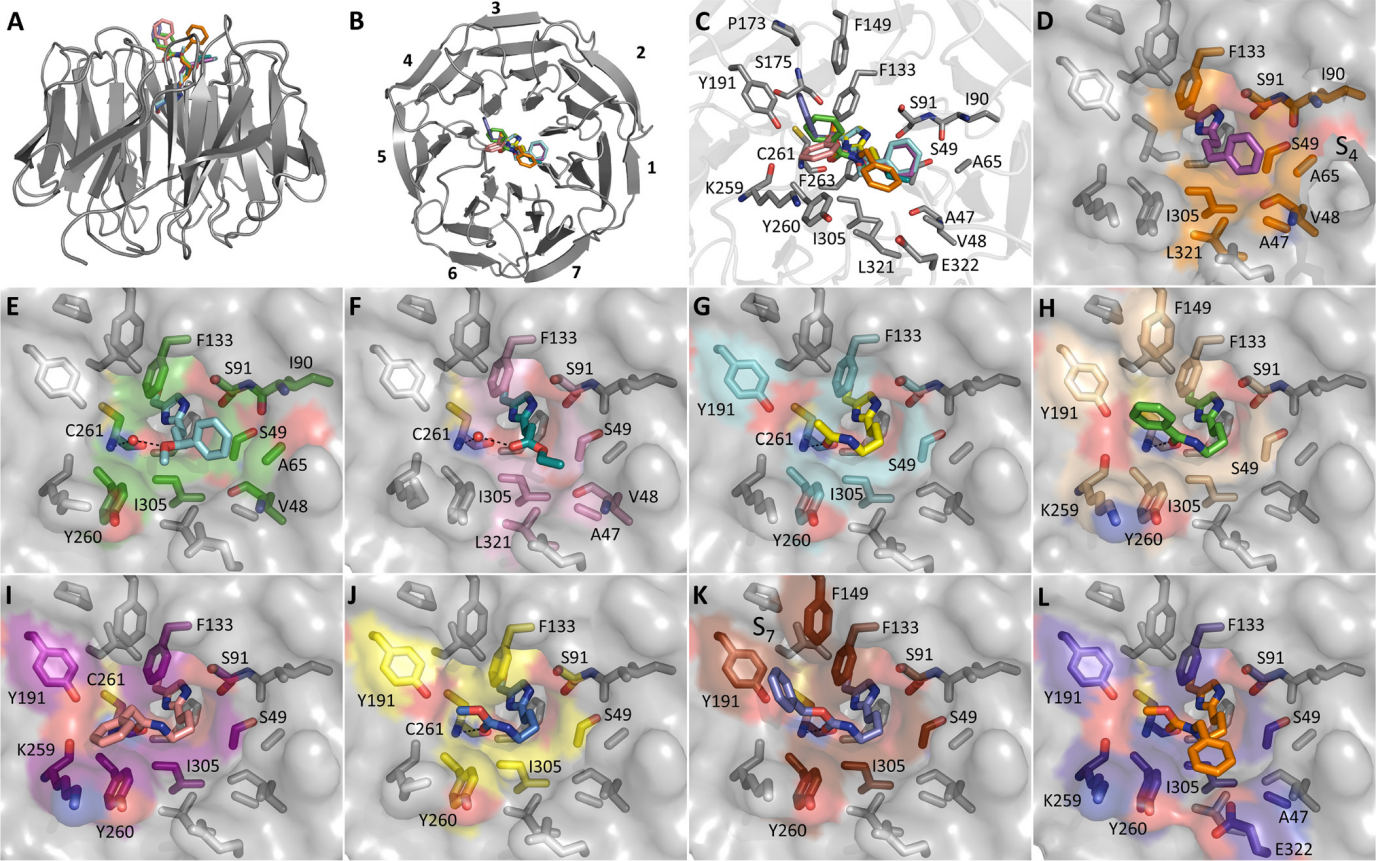
(a) Overlay of the nine complexes presented and (b) rotated by 90°. The seven blades of the WD40 domain are numbered from 1 to 7. (c) Zoomed overlay detailing nearby residues. Protein atoms displayed for A–C are from the WDR5/**35** complex. Cocrystal structures of WDR5 bound to (d) **16**, (e) **17**, (f) **24**, (g) **29**, (h) **30**, (i) **31**, (j) **32**, (k) **35**, and (l) **37**. Dashed black lines represent hydrogen bonds. WDR5 is represented with a surface depiction, and residues within 4 Å of the ligand-substituent (i.e., excluding atoms analogous to fragment **2**) are highlighted.

While the zero atom linker showed little binding (**13**, *K*_D_ = 5.2 mM), a linker length of one, two, or three methylene units was tolerated (**14**, *K*_D_ = 530 *μ*M, **15**, *K*_D_ = 300 *μ*M, and **16**, *K*_D_ = 140 *μ*M, respectively). Interestingly, the weakly binding fragment **13** is similar to the imidazole fragment hit reported by Wang *et al.*^28^ The subtle difference in orientation between the two series' imidazole rings appears key to avoid clashes with nearby residues S49 and I305. A cocrystal structure bound with **16** was solved, showing the introduced phenyl ring projecting into subpocket S4, toward residues A47, S49, A65, and L321 [Fig. 3(d)]. A methoxy substituent off the linker of **16** gave a decent improvement to binding, **17** (*K*_D_ = 22 *μ*M), ostensibly from an introduced water-mediated hydrogen bond to C261. Despite the improved binding of **17**, the initial addition of the phenyl ring was noted to constitute a sizeable drop in ligand efficiency from the initial hit (**16**, LE = 0.33; **17**, = 0.35).

**SCHEME 3.**
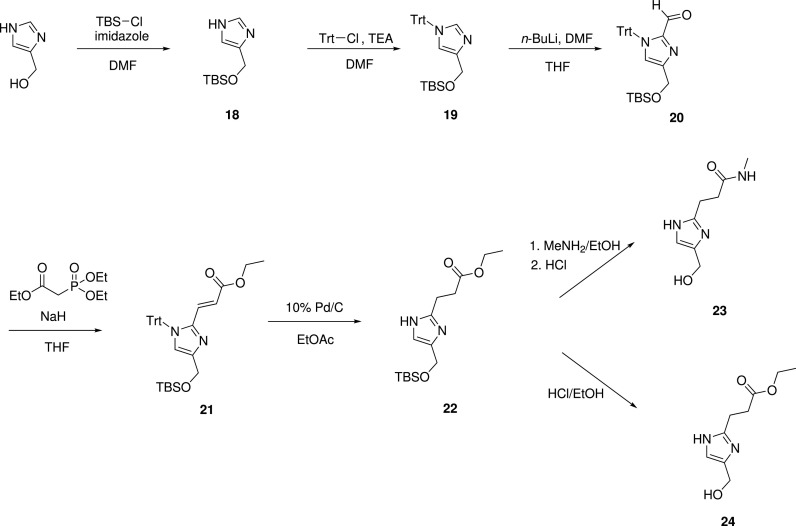
Synthesis of compounds **23** and **24**.

In an effort to improve the ligand efficiency of the 2-substituted imidazoles, two compounds that lacked the phenyl ring of **17** were synthesized (**23** and **24**, Scheme 3). These derivatives sought to retain the water-mediated hydrogen bond to C261 with a suitable hydrogen bond acceptor at a distance from the imidazole ring equivalent to the methoxy in **17** while also providing a synthetic handle for potential elaboration. While the ester-containing **24** marginally improved binding (*K*_D_ = 240 *μ*M) over the initial fragment hit, the amidic **23** lost binding (*K*_D_ = 890 *μ*M).

### C. Use of a primary amine as a synthetic handle

**SCHEME 4.**
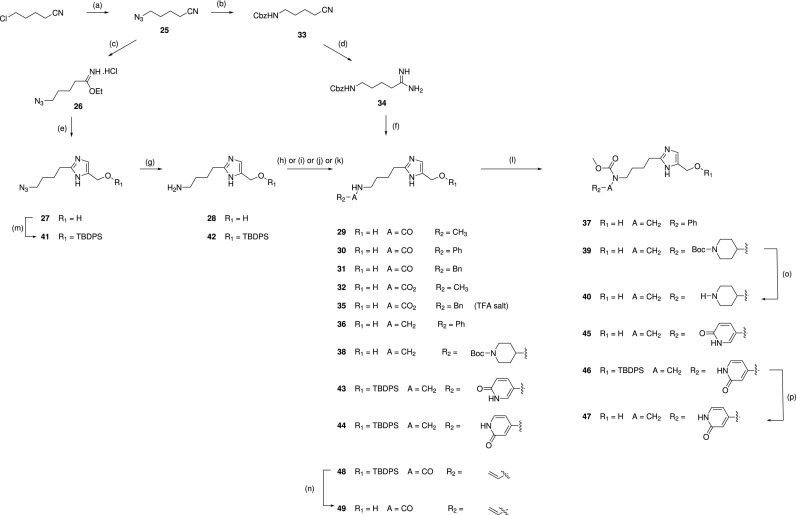
Synthesis of 4(5)-(hydroxymethyl)imidazole derivatives. Reaction conditions for the scheme: (a) NaN_3_, DMSO, 50 °C; (b) 1. Pd/C, H_2_, MeOH and 2. Cbz-Cl, Et_3_N, DCM; (c) HCl_(g)_, EtOH, 2 h; (d) 1. AcCl/EtOH and 2. NH_3_/MeOH; (e) dihydroxyacetone, NH_4_OH/*i*-PrOH, microwave, 90 °C, 1 h; (f) dihydroxyacetone, NH_3_.H_2_O; (g) Pd/C, H_2_, MeOH; (h) Ac_2_O, DCM, MeOH, rt; (i) ClCOR_2_, DCM, MeOH, Et_3_N; (j) R_2_COH, NaCNBH_3_, MeOH, AcOH; (k) ClCOR_2_, NaOH, H_2_O/DCM; (l) ClCO_2_CH_3_, DCM, TEA; (m) TBDPS-Cl, Et_3_N, DCM; (n) TBAF, THF; (o) TFA, DCM; and (p) HCl/dioxane.

In addition to the above derivatives, installation of a convenient synthetic handle onto initial hit **1** was pursued by condensing the azido-imidate **26** with dihydroxyacetone followed by a reduction to the primary amine **28** (Scheme 4). While binding of the butylamine was tolerated (**28**, *K*_D_ = 350 *μ*M), it also enabled the synthesis of a number of amides and carbamates with substituents aimed at more completely occupying the WIN binding site.

Acetylating **28** to give **29** marginally improved binding (*K*_D_ = 200 *μ*M), cocrystallization revealing a binding mode that introduces a direct hydrogen bond to Cys261, and a vector oriented toward the hydrophobic subpocket S7 [Fig. 3(g)]. Addition of hydrophobic phenyl or benzyl moieties markedly improved affinity (**30** and **31**, *K*_D_ = 12 and 25 *μ*M, respectively). The phenyl group of **30** extends to form additional interactions to F133, F149, and Y191, while the benzyl group of **31** orients away from these residues instead to interact with the sidechains of K259 and Y260 (Fig. 3). A carbamate analog of **29** (**32**, *K*_D_ = 28.9 *μ*M, LE = 0.39) provided a compound that maintained the high ligand efficiency of the original fragment hit. A similar binding mode to **29** is observed, with **32** maintaining what appears to be a key hydrogen bond to Cys261 (Fig. 3). Similar to **29** vs **31**, addition of an aromatic moiety to **32** further improved binding (**35**, *K*_D_ = 11 *μ*M), picking up interactions to F149 and Y191 (Fig. 3). Weaker electron density for the phenyl ring, relative to the rest of the molecule, suggests that this moiety maintains some mobility. Addition of the benzyl group instead to the carbamate nitrogen of **32** (**37**, *K*_D_ = 4.5 *μ*M) similarly improved binding, albeit with similarly weak electron density for the introduced ring.

### D. *N*-substituted carbamate derivatives

With the crystal structure of **32** indicating a key H-bond between the carbamate and C261, further synthetic efforts were aimed at retaining this interaction while exploring N-substituents that could project toward A65. The cocrystal structure of **37** showed the benzyl substituent in close proximity to the side chain of E322, from the para position of the phenyl ring [Fig. 3(l)]. Replacing the phenyl of **37** with a 4-piperidine (**40**) afforded a hydrogen bond donor at this position to potentially interact with E322. It was noted that such a moiety could also potentially reorient toward residue D107. Such an interaction to D107 is present in the WDR5 inhibitor OICR-9429 through a pyridone ring.^25^ Pyridone analogs of **37** were additionally thus synthesized to mimic this interaction (**45** and **47**). Unfortunately, no improvement to affinity relative to compound **37** was observed for these three compounds (**40**, **45**, and **47**). In particular, compound **45** displayed a marked loss of affinity (*K*_D_ = 98.6 *μ*M, 20-fold weaker binding than **37**), suggesting perhaps that the ring had limited scope to reorient and that electrostatic repulsion was occurring between E322 and the introduced oxygen atom of the pyridone. A study by Wang *et al.*^28^ showed that small substitutions on an aromatic ring in the S7 subpocket can deliver significant affinity gains and would suggest **35** as an attractive compound for further development although **35** currently lacks the requisite potency for a WDR5 tool compound.

### E. Probing irreversible inhibition at C261

**FIG. 4.**
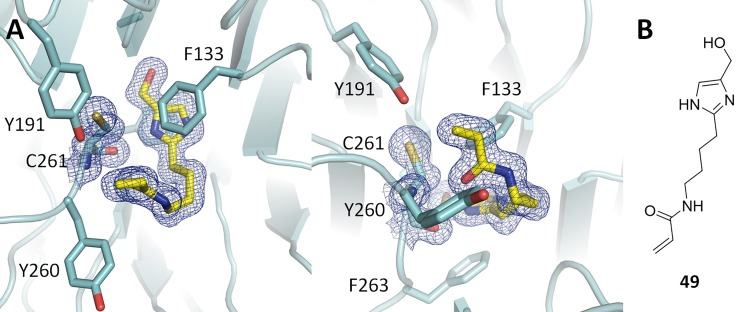
(a) Cocrystal structure of WDR5 bound to **49**. Electron density maps (2mF_o_-DF_c_, 1 sigma) are displayed for **49** and residue C261. (b) Chemical structure of **49**.

The proximity of the reported series to residue C261 suggested the potential to design irreversible inhibitors of WDR5. A compound possessing the scaffold of **29** and a reactive acrylamide headgroup (**49**) that could potentially form a covalent bond with the thiol side chain of C261 was synthesized. Compound **49** was incubated with a solution of WDR5 for 24 h at room temperature, prior to being crystallized. A high resolution (1.54 Å) structure was obtained; however, there was no evidence of covalent bonding to the protein. The cocrystal structure (Fig. 4) suggests that potentially the movement of the Cys261 side chain may be limited and that the hydrogen bond between the ligand and the amide of Cys261 is obstructing the required geometry to react with the thiol.

### F. Imidazole-bearing peptidomimetics

**FIG. 5.**
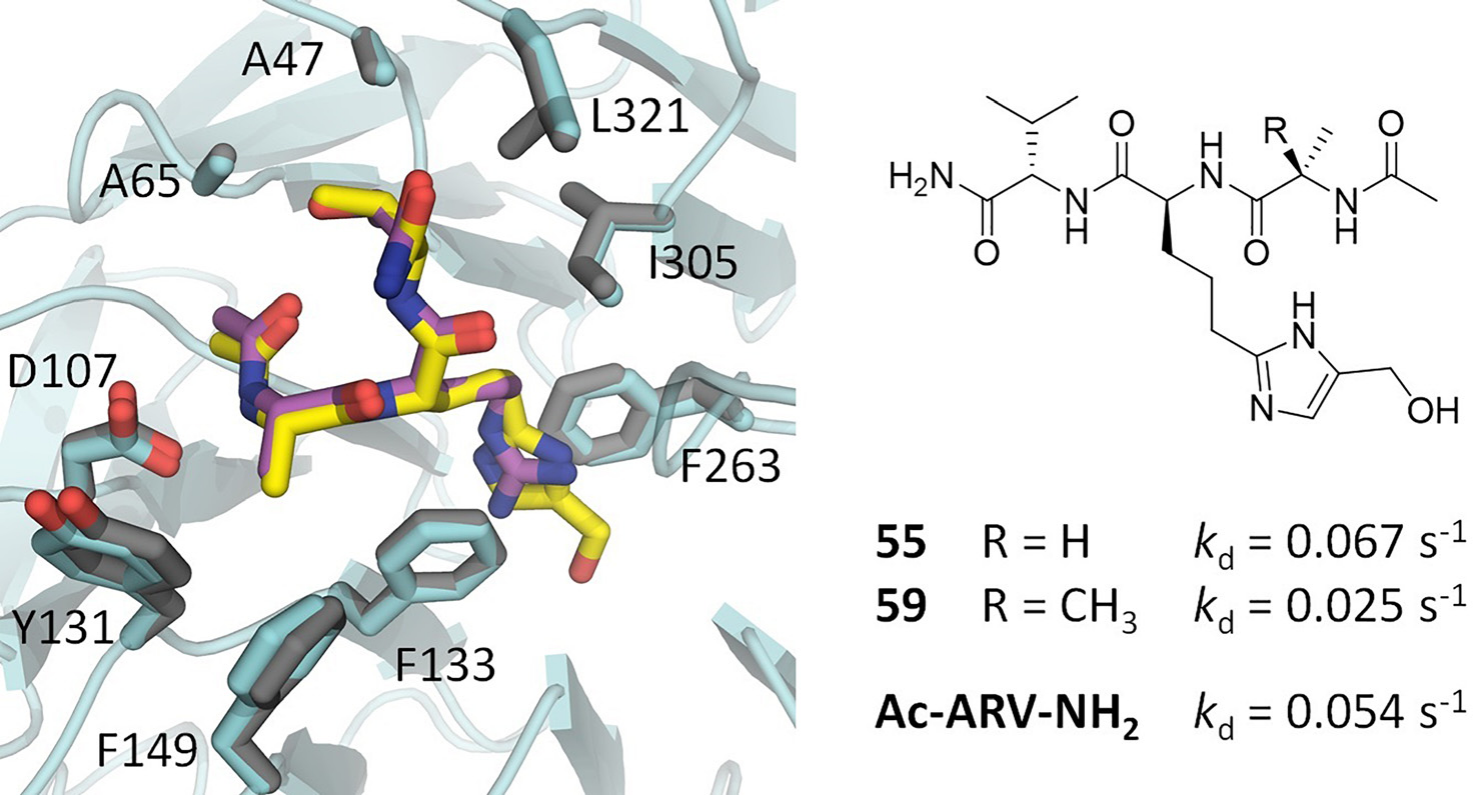
Cocrystal structures of WDR5 bound to **55** (yellow, WDR5 atoms in blue) overlaid with NαH3 peptide (purple, displayed Ac-ART-NH_2_, WDR5 atoms in grey) [3PSL].^20^ The drawn chemical structure (right) is as per the compound observed to bind in the displayed high-resolution structure (1.50 Å); however, the samples of **55** and **59** tested were a mixture of diastereomers. As the dissociation rate is concentration independent, this value is provided to allow a more meaningful comparison of binding.

Compound **1** was identified as a highly ligand efficient hit that binds the phenylalanine clamp of the WIN site, approximating the arginine headgroup of the WIN motif. The lack of net charge at physiological pH suggested this compound as a scaffold that might potentially reduce permeability issues associated with compounds possessing highly basic groups like guanidine or piperidine. Two peptidomimetics, with **1** replacing the arginine, were synthesized. Compounds **55** and **59** are analogs of the Ac-ARV-NH_2_ and Ac-α-MeAla-RV-NH_2_ tripeptides,^21^ respectively (Fig. 5). Binding was observed from both compounds to WDR5, and a cocrystal structure of **55** revealed that the ligand maintained the binding mode of the N-α-acetylated form of the H3 peptide ([3PSL],^20^ RMSD = 0.3 Å, for equivalent heavy atoms, Fig. 5). The hydroxymethyl imidazole thus appears to be a good replacement for the charged arginine residue in this context, and **59** may serve as a tool compound with more sensible physicochemical parameters than previous potent tripeptides. The high-resolution cocrystal structure of **55**/WDR5 (1.50 Å) shows unambiguous density for the ligand, and the SPR sensorgrams of **55** and **59** fit well with a 1:1 binding model (supplementary Fig. S2), suggesting a single dominant binding partner. However, epimerization during synthesis resulted in an impure mixture of diastereomers for these two compounds (supplementary Scheme S1), and the proportion of each diastereomer is unclear. Binding constants of compounds **55** and **59** are thus difficult to derive. Instead, the (concentration independent) dissociation rates have been reported for **55** and **59** (*k*_d_ = 0.067 s^−1^ and 0.025 s^−1^, respectively) to provide a more meaningful comparison to the analogous tripeptide Ac-ARV-NH_2_ (*k*_d_ = 0.054 s^−1^). Considering the known SAR of the WIN site, it would be expected that D-amino acid-bearing peptidomimetics bind WDR5 with a markedly reduced affinity. However, if multiple diastereomers of **55**/**59** were indeed binding WDR5, it would not detract from the conclusion that hydroxymethyl imidazole-peptidomimetics have potential as WDR5 WIN-site inhibitors.

## III. CONCLUSION

Recent studies indicate WDR5 as a promising cancer target beyond MLL-r cancers,^29,30^ with the potential for WDR5 inhibitors to deliver clinical impact. However, high-affinity binding to WDR5 has not necessarily delivered compounds with useful levels of methyltransferase inhibition and lower cellular proliferation.^25,28,33^ Poor cellular permeability is presumably part of the explanation, but it is unlikely to fully account for the disconnect. This study identifies a highly ligand efficient motif with multiple hydrogen bonding interactions to the WDR5 protein. This could form the basis for the design of high-affinity drug-like molecules with enhanced responses in cellular assays through further optimization.

## IV. METHODS

### A. Protein expression and purification

WDR5 sequences (WDR5^23–334^ and WDR5^32–334^) were cloned into a modified pET43 (N-terminal hexahistidine-SUMO tags) vector. Transformed *E. coli* BL21-CodonPlus-RIL cells were grown overnight in TB media supplemented with 34 *μ*g·ml^−1^ of chloramphenicol and 100 *μ*g·ml^−1^ of ampicillin for selection. The overnight culture was subcultured into TB media (2 × 1 L) and grown at 310 K to an OD_600_ ∼ 0.8 with induction by 0.1 mM isopropyl β-D-1-thiogalactopyranoside (IPTG), carried out at 289 K for 20 h. The cultures were centrifuged at 4000 × g for 10 min, and the cell pellets were frozen at 253 K. For purification, cell pellets were thawed and suspended in 200 ml lysis buffer containing 50 mM Tris, pH 8.0, 150 mM NaCl, 10 mM imidazole, 2 mM MgCl_2_, 5 mM DTT, 6.25 units·ml^−1^ Benzonase, 0.5 mg·ml^−1^ lysozyme, 1 mM PMSF, and 4 EDTA-free cOmplete™ protease-inhibitor cocktail tablets (Roche). The suspension was lysed by three passes through an ice-cooled Avestin C5 cell crusher and then centrifuged at 50 000 × g for 20 min. The supernatant was filtered (5 *μ*m) and purified via a native IMAC program on a Profinia™ (Bio-Rad), using a HiTrap IMAC FF (5 ml, GE Healthcare), being washed (10 column volumes per buffer) sequentially using buffers containing 10 mM and 20 mM imidazole (50 mM Tris, pH 8.0, 150 mM NaCl, 5 mM DTT), prior to elution with 250 mM imidazole. The eluate was further purified using gel filtration on a Superdex 200 26/60 (50 mM Tris, pH 8.0, 150 mM NaCl, 5 mM DTT). Ulp1 (SUMO) protease was added (1 U per 400 *μ*g protein) to the sample overnight at 277 K. The sample was diluted 1:1 with 50 mM Tris, pH 8.0, 150 mM NaCl, 5 mM DTT, and 40 mM imidazole (final imidazole = 20 mM), added to the IMAC column, and washed with two column volumes of buffer containing 20 mM imidazole. The flowthrough was concentrated (Amicon Ultra, 10 kDa cut-off) and then purified using a Superdex 75 16/60 (50 mM Tris, pH 8.0, 150 mM NaCl, 5 mM DTT). Protein was concentrated, dispensed as aliquots, and snap frozen. Protein identity and purity were authenticated by SDS PAGE and mass spectrometry. ∼25 mg of purified protein was obtained per liter of culture.

### B. Surface Plasmon Resonance (SPR)

SPR assay of small molecule binding used minimally biotinylated WDR5^23–334^ protein captured on streptavidin-coated SPR chips. WDR5 (1 eq.) was mixed with freshly water-dissolved EZ-link Sulfo-NHS-LC-LC-biotin (0.8 eq., Thermofisher Scientific). The reaction mixture was incubated on ice for 2 h; unreacted biotin reagent was then removed from the mixture using size-exclusion chromatography on a Superdex 75 (10/300) column equilibrated in storage buffer (10 mM HEPES, 150 mM NaCl, 5 mM DTT).

SPR experiments were performed using a Biacore T200 or S200 biosensor with a CM5 chip (GE Healthcare). Streptavidin was coupled to the chip surface at 310 K in HBS-P+ running buffer (10 mM HEPES, pH 7.4, 150 mM NaCl, 0.05%[v/v] Tween-20) after a 10 min activation with a 1:1 mixture of NHS/EDC (*N*-hydroxysuccinimide/*N*-ethyl-*N*′-(3-diethylaminopropyl)carbodiimide). Protein was diluted to 100 μg·ml^−1^ at pH 4.5 (10 mM sodium acetate, 0.005%[v/v] Tween-20) and injected for 7 min. The surface was then blocked with 1 M ethanolamine, pH 8.0, for 7 min. Immobilization levels were typically 7000–13 000 RU, but as low as 1800 RU for compounds **55, 59**, and Ac-ARV-NH_2_, where fitting uses a 1:1 kinetic model to determine dissociation rates. WDR5 capture was then performed over the streptavidin-coupled flow-cells at 293 K in HBS-P+ buffer with the addition of 5 mM DTT. Capture levels for fragment screening were ∼19 000 RU, while capture levels for subsequent testing were ∼9000 RU. The N-terminal domain of heat-shock protein 90 (Hsp90, UnprotKB P08238, residues 1–382) was captured (∼11 000 RU) during screening to provide an unrelated negative control surface, activity being monitored with intermittent ADP (adenosine diphosphate) injections. Biotin was subsequently injected over WDR5- and negative control protein-bound flow-cells to block remaining unbound streptavidin. SPR experiments were performed at 298 K in SPR running buffer containing 50 mM HEPES, 150 mM NaCl, 5 mM DTT, and 0.05%[v/v] Tween-20, 2%[v/v] DMSO. Initial fragments were screened at 100 *μ*M. When performing dose-response testing, analytes were serially diluted (2- or 3-fold), injected for 30 s contact time, and allowed to dissociate for 30 s. The flow rate was 60 *μ*l·min^−1^. Binding sensorgrams were processed, solvent-corrected, and double-referenced using Scrubber software (BioLogic Software, Australia). SPR binding analysis of most of the compounds investigated in this study revealed dissociation rates that were not sufficiently slow to allow global fitting to a kinetic binding model, for which the *k*_d_ (dissociation rate constant) must typically be <0.5 s^−1^ for SPR instruments to be able to capture sufficient data points during the dissociation phase. Therefore, to determine binding affinities (*K*_D_ values), responses at equilibrium for each analyte were fitted to a 1:1 steady-state affinity model available within Scrubber. For compounds where the top concentration tested was less than 2-fold above the *K*_D_, affinities were fit using a global Rmax against reference compounds. Sensorgrams and binding isotherms are displayed in supplementary Fig. S2.

### C. Crystallization and x-ray structure determination

Crystallization experiments were performed at the CSIRO Collaborative Crystallization Centre . The sitting-drop vapor diffusion method was used at 281 K with droplets consisting of 150–200 nl of protein solution and reservoir solution and using a reservoir volume of 50 *μ*l. Both constructs of WDR5 (WDR5^23–334^ and WDR5^32–334^) were typically used when attempting crystallization of WDR5 complexes. The protein solution was at 8.2 mg·ml^−1^ for the initial complex with **1** and at 5.1 mg·ml^−1^ for all subsequent complexes. Crystallization conditions are as follows: ammonium sulfate/ammonium fluoride, 0.024–0.237 M; sodium cacodylate/bis-tris chloride, 0.1 M, pH 5.62–7.06; PEG 3350/PEG 4000/PEG 8000/MPEG 5000, 22.1–30.5%[w/v]. Specific conditions for each complex are provided in supplementary Table S1. 1 mM of compound was added to the protein solution prior to dispensing into the plate. Compound **49** was added to the protein mix and left for 24 h at room temperature, prior to plating, to allow any potential reaction to take place that might otherwise be prevented at the low temperature (281 K) at which crystals were grown. Crystals were harvested from drops at 277 K and cryoprotected in 30%[v/v] ethylene glycol. Diffraction data were collected at the MX1^34^ and MX2^35^ beamlines at the Australian Synchrotron. Datasets were indexed using XDS^36^ or Dials^37^ and scaled using Aimless.^38^ Molecular replacement was performed using Phaser^39^ with 2H9L^32^ as a reference structure. Refinement was performed using Refmac^40^ and Coot^41^ with ligands' geometric restraints generated using Phenix eLBOW^42^ or PRODRG.^43^ A summary of data collection and refinement statistics is given in Table I, with full details in supplementary Table S1. Difference density maps for ligands are given in supplementary Fig. S1.

### D. Compound synthesis and characterization

Unless otherwise stated, the following generalizations apply. ^1^H NMR spectra were recorded on a Bruker Ultrashield Plus (400 MHz) or a Bruker AVANCE III (400 MHz). The multiplicity of a signal is designated by the following abbreviations: s, singlet; d, doublet; t, triplet; q, quartet; dd, doublet of doublets; dt, doublet of triplets; tt, triplet of triplets; br, broad; and m, multiplet. All observed coupling constants, *J*, are reported in Hertz. Exchangeable protons are not always observed.

LCMS (liquid chromatography-mass spectrometry) data were generated using the conditions described below. Chlorine isotopes are reported as ^35^Cl, and bromine isotopes are reported as either ^79^Br or ^81^Br or both ^79^Br/^81^Br.

#### 1. LCMS method A (LCMS-A)

Instrument: Agilent 6100 Series Single Quad LC/MS and Agilent 1200 Series HPLC. Pump: 1200 Series G1311A Quaternary pump. Autosampler: 1200 Series G1329A Thermostatted Autosampler. Detector: 1200 Series G1314B Variable Wavelength Detector. Column: Luna C8 (2) 5 *μ*m 50 × 4.6 mm 100 Å. Column temperature: 30 °C. Injection volume: 5 *μ*l. Solvent A: Water 0.1% Formic Acid. Solvent B: MeCN 0.1% Formic Acid. Gradient: 5%–100% solvent B over 10 min. Detection: 254 nm or 214 nm. MS Conditions: Ion Source: Quadrupole. Ion Mode: Multimode-ES. Drying gas temp: 300 °C. Vaporizer temperature: 200 °C. Capillary voltage (V): 2000 (positive). Capillary voltage (V): 4000 (negative). Scan range: 100–1000. Step size: 0.1 s. Acquisition time: 10 min.

#### 2. LCMS method B (LCMS-B)

Instrument: Agilent 1260 Infinity Series UPLC/MS. Pump: 1260 Infinity G1312B Binary Pump. Autosampler: 1260 Infinity G1367E 1260 HiP ALS. Detector: 1290 Infinity G4212A 1290 DAD. Column: Poroshell 120 EC-C18 2.7 *μ*m 50 × 3.0 mm. Column temperature: 35 °C. Injection volume: 1 *μ*l. Solvent A: water 0.1% Formic Acid. Solvent B: MeCN 0.1% Formic Acid. Gradient: 5%–100% solvent B over 3.8 min. Detection: monitored at 254 nm and 214 nm. MS conditions: Ion Source: Quadrupole. Ion Mode: API-ES. Drying gas temp: 350 °C. Capillary voltage (V): 3000 (positive). Capillary voltage (V): 3000 (negative). Scan range: 100–1000. Step size: 0.1 s. Acquisition time: 5 min.

#### 3. LCMS method C (LCMS-C)

Instrument: Agilent Technologies 1290 series. Pump type: binary pump. Detector type: Diode Array Detector. Column: Agilent Poroshell 120 EC-C18, 2.7 *μ*m, 4.6 × 50 mm. Column temperature: 35 °C. Acquisition wavelengths: 214 nm and 254 nm. Mobile phase: A: 0.05% HCOOH in water (v/v) and B: 0.05% HCOOH in Acetonitrile (v/v). Run time: 5 min. Flow rate: 1.0 ml/min. MS model: Agilent G6120A, quadrupole LC/MS. Ion source: ES-API (+ve or −ve). TIC: 70 ∼ 1000 m/z. Fragmentor: 60. Drying gas flow: 10 l/min. Nebulizer pressure: 35 psi. Drying gas temperature: 350 °C. Vcap: 3000 V.

#### 4. LCMS method D (LCMS-D)

Instrument: Agilent 6120 Series Single Quad LC/MS and Agilent 1200 Series HPLC. Pump: 1200 Series G1311A Quaternary pump. Autosampler: 1200 Series G1329A Thermostatted Autosampler. Detector: 1200 Series G1314B Variable Wavelength Detector. Column: Luna C8 (2) 5 *μ*m 50 × 4.6 mm 100 Å. Column temperature: 30 °C. Solvent A: Water 0.1% Formic Acid. Solvent B: MeCN 0.1% Formic Acid. Gradient: 0%–95% solvent B over 10 min. Detection: 254 nm or 214 nm. MS conditions: Ion source: quadrupole. Ion mode: Multimode-ES & APCI. Drying gas temp: 250 °C. Vaporizer temperature: 200 °C. Capillary voltage (V): 4000 (positive). Capillary voltage (V): 4000 (negative). Scan range: 100–1000. Step size: 0.1 s. Acquisition time: 10 min.

Microwave irradiation was performed using a CEM Explorer SP Microwave Reactor.

Where necessary, anhydrous solvents were purchased from Sigma-Aldrich or dried using conventional methods. Solutions of inorganic acids or bases were made up as aqueous solutions unless stated otherwise.

Analytical thin-layer chromatography was performed on Merck silica gel 60 F254 aluminium-backed plates that were visualized using fluorescence quenching under UV light or a basic KMnO_4_ dip or Ninhydrin dip.

Preparative thin-layer chromatography (prep TLC) was performed using Tklst (China), grand grade: (HPTLC): 8 ± 2 *μ*m > 80%; (TLC): 10–40 *μ*m. Type: GF254. Compounds were visualized by UV (254 nm).

Flash chromatography was performed using a Biotage Isolera purification system using either Grace or RediSep^®^ silica cartridges.

Column chromatography was performed using Tklst (China), grand grade, 100–200 meshes silica gel.

Cartridge details: *SCX and SCX-2 cartridges:* Manufacturer: Biotage. Product: ISOLUTE^®^ SCX 1 g, (6 ml SPE Column unless otherwise stated); manufacturer: Biotage. Product: ISOLUTE^®^ SCX-2 1 g (6 ml Column).

### E. 4-(chloromethyl)-2-methyl-1*H*-imidazole hydrochloride 12

A solution of (2-methyl-1*H*-imidazol-4-yl)methanol hydrochloride (117 mg, 0.787 mmol) and thionyl chloride (1 ml) was stirred at room temperature overnight. Chloroform (1 ml) was added, and the mixture was concentrated *in vacuo* to give the title compound as a brown oil (144 mg, ∼80% purity, ∼88% yield). The material was used in the next step without further purification. ^1^H NMR (400 MHz, DMSO-d_6_) δ 7.59 (s, 1H), 4.84 (s, 2H), 2.56 (s, 3H). ^1^H NMR data matched the reported values.^44^

### F. 4-methoxy-2-methyl-1*H*-imidazole hydrochloride 7

A solution of 4-(chloromethyl)-2-methyl-1*H*-imidazole hydrochloride **12** (34.5 mg, 0.207 mmol) and methanol (1 ml) was heated at reflux for 2 h and then irradiated in a microwave at 150 °C for 1 h. The mixture was returned to room temperature, concentrated *in vacuo*, dissolved in a minimum volume of MeOH, and loaded onto an SCX cartridge. The cartridge was washed with MeOH (10 ml) before the product was eluted with 7 M NH_3_ in MeOH (10 ml). The eluate was concentrated *in vacuo* and then further purified by column chromatography (4 g SiO_2_ cartridge, 1%–15% MeOH (with 10% 7 M NH_3_ in MeOH) in DCM) to give the title compound as a colorless solid (4.5 mg, 17%): ^1^H NMR (400 MHz, CDCl_3_) δ 6.87 (s, 1H), 4.38 (s, 2H), 3.37 (s, 3H), 2.40 (s, 3H).

### G. General procedure for synthesis of 8–11

A solution of 4-(chloromethyl)-2-methyl-1*H*-imidazole hydrochloride **12** (30 mg, 0.180 mmol) and alcohol (1 ml) was irradiated in a microwave at 150 °C for 15 min. The solution was returned to room temperature and concentrated *in vacuo*. The residue was dissolved in a minimum volume of MeOH and loaded onto an SCX cartridge, washed with MeOH (10 ml), and then eluted with 7 M NH_3_ in MeOH (10 ml). The eluate was concentrated *in vacuo* and the residue was purified by column chromatography (4 g SiO_2_ cartridge, 5–15% MeOH (with 10% 7 M NH_3_ in MeOH) in DCM) to give the title compound (see below).

2-methyl-4-(propoxymethyl)-1*H*-imidazole **8**: 6.8 mg, 24% yield. ^1^H NMR (400 MHz, chloroform-d) δ 6.88 (s, 1H), 5.79 (br s, 1H), 4.42 (s, 2H), 3.43 (t, J = 6.8 Hz, 2H), 2.43 (s, 3H), 1.68–1.52 (m, 2H), 0.90 (t, J = 7.4 Hz, 3H).

4-(isopropoxymethyl)-2-methyl-1*H*-imidazole **9**: 8.4 mg, 30% yield. ^1^H NMR (400 MHz, chloroform-*d*) δ 6.84 (s, 1H), 4.43 (d, J = 0.7 Hz, 2H), 3.71 (hept, J = 6.1 Hz, 1H), 2.39 (s, 3H), 1.19 (d, J = 6.1 Hz, 6H).

2-methyl-4-((2,2,2-trifluoroethoxy)methyl)-1*H*-imidazole **10**: 12.5 mg, 35% yield. ^1^H NMR (400 MHz, chloroform-d) δ 6.93 (s, 1H), 4.60 (s, 2H), 3.87 (q, J = 8.8 Hz, 2H), 2.42 (s, 3H). ^19^F NMR (376 MHz, chloroform-d) δ -73.83.

4-((benzyloxy)methyl)-2-methyl-1*H*-imidazole **11**: 8.7 mg, 23% yield. ^1^H NMR (400 MHz, chloroform-*d*) δ 7.36 – 7.26 (m, 5H), 6.86 (s, 1H), 4.56 (s, 2H), 4.47 (d, J = 0.7 Hz, 2H), 2.38 (s, 3H).

### H. General procedure for synthesis of 14 and 15

A suspension of dihydroxyacetone (50 mg, 0.55 mmol) and a commercially available amidine hydrochloride (0.555 mmol) in ammonium hydroxide 33%–35% (0.5 ml) was stirred in the microwave at 120 °C for 10 min. The reaction mixture was evaporated to dryness and loaded onto an SCX-2 cartridge (0.5 g). The cartridge was washed with MeOH (20 ml) before the product was eluted with 3.5 M NH_3_ in MeOH (10 ml). The basic fraction was concentrated under reduced pressure, and the resultant dark oil was triturated with acetone (∼10 ml). The oil was dried under high vacuum to yield the desired product.

(2-benzyl-1*H*-imidazol-4-yl)methanol **14**: ^1^H NMR (400 MHz, Methanol-*d*_4_) δ 7.31 – 7.18 (m, 7H), 6.87 (s, 1H), 4.50 (d, *J *=* *0.8 Hz, 2H), 4.01 (s, 2H).

(2-phenethyl-1*H*-imidazol-4-yl)methanol **15**: ^1^H NMR (400 MHz, Methanol-*d*_4_) δ 7.27 – 7.21 (m, 2H), 7.18 – 7.13 (m, 3H), 6.83 (s, 1H), 4.50 (d, *J *=* *0.8 Hz, 2H), 3.02 – 2.90 (m, 4H).

### I. (2-(3-phenylpropyl)-1*H*-imidazol-4-yl)methanol 16

#### 1. Step 1: 4-phenylbutanimidamide 60

Under an inert atmosphere, acetyl chloride (0.59 ml, 8.3 mmol) was added to a solution of 4-phenylbutyronitrile (0.150 g, 1.03 mmol) in anhydrous EtOH (1.21 ml) and the mixture was stirred at room temperature for 5 h. The volatiles were removed *in vacuo*, and the solid residue was stirred in 2 M NH_3_ in EtOH (2.5 ml) for 16 h at room temperature. The mixture was filtered, the filtrate was concentrated *in vacuo*, and the resultant residue was purified by column chromatography (Biotage Isolera, 12 g SiO_2_ cartridge, 0%–20% MeOH in DCM) to give the title compound as a white solid (0.081 g, 48%). The product had a weak chromophore and was unsuitable for analysis by LCMS. ^1^H NMR (400 MHz, DMSO) 8.80 (br s, 3H), 7.34 – 7.27 (m, 2H), 7.24 – 7.15 (m, 3H), 2.64 – 2.57 (m, 2H), 2.43 – 2.37 (m, 2H), 1.96 – 1.85 (m, 2H).

#### 2. Step 2: (2-(3-phenylpropyl)-1*H*-imidazol-4-yl)methanol 16

A mixture of 4-phenylbutanimidamide **60** (0.081 g, 0.50 mmol) and dihydroxyacetone (0.045 g, 0.50 mmol) in 28%–30% ammonia solution (1 ml) was stirred in the microwave at 120 °C for 10 min. The solvent was evaporated *in vacuo*, and the residue was dissolved in MeOH and loaded onto an SCX-2 cartridge (500 mg). The cartridge was washed with 3 column volumes of MeOH before the desired product was eluted with 3 column volumes of 7 M NH_3_ in MeOH. The basic fraction was concentrated *in vacuo* and purified further by column chromatography (Biotage Isolera, 4 g SiO_2_ cartridge, 0%–20% MeOH (containing 10% 7 M NH_3_/MeOH solution) in DCM). Fractions containing the desired product were combined and purified further by mass directed preparative LCMS (5% –> 95% MeCN/0.1% TFA in H_2_O/0.1% TFA) to give the title compound as an orange oil (0.006 g, 6%). LCMS-B: rt 2.6 min; m/z 217.0 [M + H]^+^. The product had no significant chromophore at 254 nm or 214 nm. ^1^H NMR (400 MHz, DMSO) δ 13.89 (br s, 1H), 7.35 (s, 1H), 7.32 – 7.27 (m, 2H), 7.23 – 7.19 (m, 3H), 5.62 – 5.45 (m, 1H), 4.45 (d, J = 4.5 Hz, 2H), 2.87 (t, J = 7.7 Hz, 2H), 2.61 (t, J = 7.6 Hz, 2H), 2.01 (p, J = 7.7 Hz, 2H).

### J. (2-(3-methoxy-3-phenylpropyl)-1*H*-imidazol-4-yl)methanol 17

#### 1. Step 1: 4-methoxy-4-phenylbutanimidamide 61

Under an inert atmosphere, acetyl chloride (0.75 ml, 11 mmol) was added to a solution of 4-methoxy-4-phenylbutanenitrile (0.230 g, 1.31 mmol) in anhydrous EtOH (1.53 ml), and the mixture was stirred at room temperature for 5 h. The volatiles were removed *in vacuo*, and the solid residue was stirred in 2 M NH_3_ in EtOH (2.5 ml) for 16 h at room temperature. The mixture was filtered, and the filtrate was concentrated *in vacuo* to give the title compound as a white solid (0.219 g, 87%). The product had a weak chromophore and was unsuitable for analysis by LCMS. ^1^H NMR (400 MHz, DMSO) δ 9.11 – 8.47 (m, 3H), 7.41 – 7.36 (m, 2H), 7.33 – 7.27 (m, 3H), 4.16 (dd, J = 8.3, 4.6 Hz, 1H), 3.12 (s, 3H), 2.56 – 2.47 (m, peak obscured by solvent), 2.46 – 2.36 (m, 1H), 2.05 – 1.88 (m, 2H).

#### 2. Step 2: (2-(3-methoxy-3-phenylpropyl)-1H-imidazol-4-yl)methanol 17

A mixture of 4-methoxy-4-phenylbutanimidamide **61** (0.219 g, 1.14 mmol) and dihydroxyacetone (0.103 g, 1.14 mmol) in 28%–30% ammonia solution (2 ml) was stirred in the microwave at 120 °C for 10 min. The solvent was evaporated *in vacuo*, and the residue was adsorbed onto silica and purified by column chromatography (Biotage Isolera, 12 g SiO_2_ cartridge, 0%–20% MeOH (+10% 7 M NH_3_ in MeOH) in DCM) to give an orange oil. The oil was triturated with Et_2_O before the solid was dissolved in MeOH and loaded onto an SCX-2 cartridge (500 mg). The cartridge was washed with 3 column volumes of MeOH before the desired product was eluted with 3 column volumes of 7 M NH_3_ in MeOH. The basic fraction was concentrated *in vacuo* to give the title compound as an orange oil (0.028 g, 10%). The product could not be characterized by LMCS due to the lack of a chromophore at 214 nm and 254 nm. ^1^H NMR (400 MHz, DMSO) δ 11.79 – 11.29 (m, 1H), 7.41 – 7.34 (m, 2H), 7.32 – 7.25 (m, 3H), 6.82 – 6.48 (m, 1H), 4.99 – 4.56 (m, 1H), 4.29 (s, 2H), 4.18 (dd, J = 7.4, 5.6 Hz, 1H), 3.12 (s, 3H), 2.63 – 2.51 (m, peaks obscured by solvent), 2.06 – 1.86 (m, 2H).

### K. Synthesis of compounds 23 and 24

#### 1. Intermediates

##### a. Step 1: 4-(((*tert*-butyldimethylsilyl)oxy)methyl)-1H-imidazole **18**

TBSCl (3.38 g, 22.4 mmol) was added to a stirring solution of (1*H*-imidazol-4-yl)methanol (2.00 g, 20.4 mmol) and imidazole (2.78 g, 40.8 mmol) in DMF (*N,N*-dimethylformamide) (50 ml) under N_2_ at 0 °C. The mixture was allowed to slowly reach room temperature overnight. A saturated solution of NaHCO_3_ (50 ml) was added, and the mixture was stirred for 1 h. The mixture was extracted with EtOAc (3 × 100 ml), and the combined organic layers were washed with water (3 × 100 ml) and brine (100 ml), dried (Na_2_SO_4_), and concentrated *in vacuo* to give a yellow/orange oil. The material was purified by column chromatography (120 g SiO_2_ cartridge, 2%–10% methanol in DCM) to give the title compound as a colorless oil (3.0 g, 69%). ^1^H NMR (400 MHz, Chloroform-d) δ 7.59 (s, 1H), 6.94 (s, 1H), 4.73 (s, 2H), 0.91 (s, 9H), 0.08 (s, 6H). LCMS-B rt 2.89 min; (m/z) 213 [M + H]^+^.

##### b. Step 2: 4-(((*tert*-butyldimethylsilyl)oxy)methyl)-1-trityl-1H-imidazole **19**

Trityl chloride (1.61 g, 5.78 mmol) was added to a solution of 4-(((*tert*-butyldimethylsilyl)oxy)methyl)-1*H*-imidazole **18** (1.12 g, 5.26 mmol) and triethylamine (1.47 ml, 10.5 mmol) in anhydrous DMF (20 ml) under a nitrogen atmosphere. The mixture was stirred for 2 h before water was added. The aqueous phase was extracted with DCM (3 × 100 ml), and the organics were combined and washed with brine (3 × 100 ml). The organic extract was dried (Na_2_SO_4_), and the solvent was removed *in vacuo*. Purification by column chromatography (Biotage Isolera, 40 g SiO_2_ cartridge, 0%–30% EtOAc in petroleum benzine 40–60 °C) gave the title compound as a white solid (1.93 g, 81%). ^1^H NMR (400 MHz, CDCl_3_) δ 7.37 (d, J = 1.5 Hz, 1H), 7.34 – 7.30 (m, 9H), 7.17 – 7.13 (m, 6H), 6.75 – 6.69 (m, 1H), 4.68 (d, J = 0.9 Hz, 2H), 0.84 (s, 9H), 0.03 (s, 6H). LCMS-B: rt 3.48 min; m/z 243.0 [Trt]^+^. Only trityl cation was observed in the mass spec.

##### c. Step 3: 4-(((*tert*-butyldimethylsilyl)oxy)methyl)-1-trityl-1H-imidazole-2-carbaldehyde **20**

A solution of 4-(((*tert*-butyldimethylsilyl)oxy)methyl)-1-trityl*-1H*-imidazole **19** (0.211 g, 0.464 mmol) in anhydrous THF (5 ml) under an atmosphere of nitrogen was cooled to −40 °C. To this solution, *n*-butyllithium (1.54 M in hexanes, 0.60 ml, 0.93 mmol) was added, and the mixture was stirred at −40 °C for 30 min. Anhydrous DMF (0.14 ml, 1.9 mmol) was added, and the mixture was returned to room temperature and stirred for 30 min. A saturated aqueous solution of NH_4_Cl was added (∼20 ml), and the aqueous phase was extracted with EtOAc (3 × 20 ml). The organics were combined, washed with brine, and dried (Na_2_SO_4_), and the solvent was removed *in vacuo*. The crude solid was purified by column chromatography (Biotage Isolera, 24 g SiO_2_ cartridge, 0%–30% EtOAc in petroleum benzine 40–60 °C) to give the title compound as a white solid (0.190 g, 85%). LCMS-B: rt 4.02 min; m/z 243.0 [Trt]^+^. Only the trityl protecting group ion could be observed in the mass spectrum. ^1^H NMR (400 MHz, CDCl_3_) 9.13 (s, 1H), 7.35 – 7.31 (m, 9H), 7.15 – 7.11 (m, 6H), 6.93 (s, 1H), 4.77 – 4.74 (m, 2H), 0.82 (s, 9H), 0.03 (s, 6H).

##### d. Step 4: Ethyl (Z)-3-(4-(((*tert*-butyldimethylsilyl)oxy)methyl)-1-trityl*-1H*-imidazol-2-yl)acrylate and ethyl (E)-3-(4-(((*tert*-butyldimethylsilyl)oxy)methyl)-1-trityl*-1H*-imidazol-2-yl)acrylate 21

Sodium hydride (60% dispersion in mineral oil, 0.116 g, 2.90 mmol) was stirred in anhydrous THF (5 ml) at room temperature under nitrogen. The mixture was cooled to 0 °C before a solution of triethyl 2-phosphonoacetate (0.650 g, 2.90 mmol) in anhydrous THF (5 ml) was added. The mixture was returned to room temperature. A suspension of 4-(((*tert*-butyldimethylsilyl)oxy)methyl)-1-trityl*-1H*-imidazole-2-carbaldehyde **20** (0.700 g, 1.45 mmol) in anhydrous THF (10 ml) was then added, and the mixture was stirred at room temperature for 24 h. Water (∼50 ml) was added and THF was removed under reduced pressure. The aqueous layer was extracted with EtOAc (3 × 50 ml), the organics were combined, washed with brine, and dried (Na_2_SO_4_), and the solvent was removed *in vacuo*. The crude oil was purified by column chromatography (Biotage Isolera, 40 g SiO_2_ cartridge, 0%–30% EtOAc in petroleum benzine 40–60 °C) to give two components (in order of elution):

(a) ethyl (E)-3-(4-(((*tert*-butyldimethylsilyl)oxy)methyl)-1-trityl*-1H*-imidazol-2-yl)acrylate **21** - 0.479 g, 60% yield. ^1^H NMR (400 MHz, CDCl_3_) δ 7.36 – 7.29 (m, 9H), 7.17 – 7.10 (m, 6H), 6.78 (s, 1H), 6.65 (d, J = 15.4 Hz, 1H), 6.49 (d, J = 15.5 Hz, 1H), 4.71 (s, 2H), 3.99 (q, J = 7.1 Hz, 2H), 1.12 (t, J = 7.1 Hz, 3H), 0.83 (s, 9H), 0.03 (s, 6H); LCMS-B rt 4.12 min; m/z 243.0 [Trt]^+^. Assigned as the (E) isomer on the basis of the alkenyl hydrogen signals: larger coupling constant and further downfield compared to the alkenyl hydrogens of the (Z)-isomer.
(b) ethyl (Z)-3-(4-(((*tert*-butyldimethylsilyl)oxy)methyl)-1-trityl*-1H*-imidazol-2-yl)acrylate - 0.152 g, 19% yield. ^1^H NMR (400 MHz, CDCl_3_) δ 7.33 – 7.29 (m, 9H), 7.19 – 7.14 (m, 6H), 6.69 (s, 1H), 5.64 (d, J = 11.9 Hz, 1H), 5.37 (d, J = 11.9 Hz, 1H), 4.66 (s, 2H), 4.17 (q, J = 7.2 Hz, 2H), 1.24 (t, J = 7.1 Hz, 3H), 0.82 (s, 9H), 0.02 (s, 6H); LCMS-B: rt 3.69 min; m/z 243.0 [Trt]^+^. Assigned as the (Z) isomer: the alkenyl hydrogens have a smaller coupling constant and are further upfield compared to the alkenyl hydrogens of **21**.

### L. 3-(4-(hydroxymethyl)*-1H*-imidazol-2-yl)-N-methylpropanamide 23

#### 1. Step 1: ethyl 3-(4-(((*tert*-butyldimethylsilyl)oxy)methyl)*-1H*-imidazol-2-yl)propanoate 22

A mixture of ethyl (E)-3-(4-(((*tert*-butyldimethylsilyl)oxy)methyl)-1-trityl*-1H*-imidazol-2-yl)acrylate **21** (0.479 g, 0.867 mmol) and Pd 10% on carbon (wetted with ca. 55% water, 0.075 g) in EtOAc was stirred under an atmosphere of hydrogen at room temperature for 21 h. The mixture was filtered through a plug of Celite, and the filtrate was concentrated under reduced pressure. The resultant residue was purified by column chromatography (Biotage Isolera, 24 g SiO_2_ cartridge, 0%–100% EtOAc in petroleum benzine 40–60 °C) to give the title compound as a colorless oil (0.144 g, 53% yield). ^1^H NMR (400 MHz, CDCl_3_) δ 6.80 – 6.75 (m, 1H), 4.66 (d, J = 0.9 Hz, 2H), 4.16 (q, J = 7.1 Hz, 2H), 3.04 – 2.99 (m, 2H), 2.75 – 2.70 (m, 2H), 1.26 (t, J = 7.1 Hz, 3H), 0.91 (s, 9H), 0.08 (s, 6H). LCMS-B RT 3.06 min; m/z 313.0 [M + H]^+^ (product had no absorption at 254 nm).

#### 2. Step 2: 3-(4-(hydroxymethyl)*-1H*-imidazol-2-yl)-N-methylpropanamide 23

A solution of ethyl 3-(4-(((*tert*-butyldimethylsilyl)oxy)methyl)*-1H*-imidazol-2-yl)propanoate (**22**) (0.018 g, 0.058 mmol) in methylamine solution (33 wt. % in absolute ethanol, 5 ml) was stirred at 50 °C for 24 h. The volatiles were evaporated under reduced pressure, and the residue was dissolved in MeOH (2 ml). Hydrochloric acid (37%, 0.05 ml) was added, and the solution was stirred at 40 °C for 6 h. The volatiles were removed under reduced pressure, and the residue was taken up in a minimal amount of MeOH and loaded onto an SCX-2 cartridge (500 mg). The cartridge was washed with 5 column volumes of MeOH before the desired product was eluted with 7 N NH_3_ in MeOH. The basic fractions were combined, and the solvent was removed *in vacuo* to give the title compound as a pale yellow oil (0.011 g, >95% yield). ^1^H NMR (400 MHz, DMSO) δ 7.87 – 7.77 (m, 1H), 6.70 (s, 1H), 4.79 (s, 1H), 4.30 (s, 2H), 2.81 – 2.73 (m, 2H), 2.56 (d, J = 4.6 Hz, 3H), 2.47 – 2.40 (m, 2H), imidazole N-H not observed; LCMS-B rt 0.45 min; m/z 184.0 [M + H]^+^.

### M. ethyl 3-(4-(hydroxymethyl)*-1H*-imidazol-2-yl)propanoate 24

A mixture of ethyl 3-(4-(((*tert*-butyldimethylsilyl)oxy)methyl)*-1H*-imidazol-2-yl)propanoate **22** (0.028 g, 0.090 mmol), HCl (37%, 0.02 ml), and EtOH was stirred at room temperature for 23 h. The volatiles were removed *in vacuo*, and the residue was taken up in MeOH and loaded onto an SCX-2 cartridge (500 mg). The cartridge was washed with 5 column volumes of MeOH, and then the product was eluted with 7 N NH_3_ in MeOH. The basic fractions were combined, and the solvent was removed under reduced pressure to give the title compound as a colorless oil (0.004 g, 23% yield): ^1^H NMR (400 MHz, MeOD) 6.86 (s, 1H), 4.51 – 4.46 (m, 2H), 4.11 (q, J = 7.1 Hz, 2H), 2.99 – 2.92 (m, 2H), 2.78 – 2.69 (m, 2H), 1.22 (t, J = 7.1 Hz, 3H); N-H and O-H not observed; the signal at 3.66 ppm indicates a small amount of methyl ester contamination; LCMS-B RT 0.46 min; m/z 199.0 [M + H]^+^; the product had no absorption at 254 nm and eluted with the solvent front.

### N. (2-(4-aminobutyl)*-1H*-imidazol-5-yl)methanol bis trifluoroacetic acid 28

#### 1. Step 1: 5-azidopentanenitrile 25

To a solution of 5-chloropentanenitrile (25.0 g, 213 mmol) in DMSO (200 ml) was added NaN_3_ (27.4 g, 425 mmol), and the mixture was heated at 50 °C overnight. The mixture was diluted with water (200 ml) and extracted with DCM (200 ml × 3). The combined organic extracts were washed with brine (200 ml), dried over Na_2_SO_4_, filtered, and concentrated under reduced pressure to give the crude title compound (38.3 g), which was used directly in the next step without further purification. LCMS-C: m/z 125.1 [M + H]^+^.

#### 2. Step 2: ethyl 5-azidopentanimidate hydrochloride 26

A solution of 5-azidopentanenitrile **25** (20.2 g) in EtOH (200 ml) at 0 °C was bubbled with HCl gas for 3 h. The solvent was removed under reduced pressure to give the crude title compound (48.7 g), which was used directly in the next step without further purification. LCMS-C: m/z 171.1 [M + H]^+^.

#### 3. Step 3: (2-(4-azidobutyl)*-1H*-imidazol-5-yl)methanol 27

A mixture of ethyl 5-azidopentanimidate hydrochloride 26 (2.4 g) and 1,3-dihydroxypropan-2-one (1.14 g, 12.7 mmol) in concentrated aqueous NH_4_OH (6 ml) and i-PrOH (6 ml) was heated at 90 °C under microwave irradiation for 1 h. This procedure was performed 20 times, and the reaction mixtures were combined and concentrated under reduced pressure. The residue was purified by silica gel chromatography (DCM/MeOH = 50:1 to 30:1 to 15:1) to give the title compound (13.8 g) as a yellow oil. LCMS-C: m/z 196.1 [M + H]^+^.

#### 4. Step 4: (2-(4-aminobutyl)*-1H*-imidazol-5-yl)methanol bistrifluoroacetic acid 28

A mixture of (2-(4-azidobutyl)*-1H*-imidazol-5-yl)methanol **27** (100 mg, 0.51 mmol) and 10% Pd/C (20 mg) in MeOH (4 ml) was stirred at room temperature under an atmosphere of hydrogen overnight. The mixture was filtered, and the filtrate was concentrated under reduced pressure. The residue was purified by reverse phase chromatography (Biotage, C18 column, eluting with MeCN in water containing 0.1% TFA) to give the title compound (56 mg, 28%). LCMS-C: m/z 170.2 [M + H]^+^. ^1^H NMR (400 MHz, MeOD) δ 7.31 (s, 1H), 4.61 (s, 2H), 3.04 – 2.96 (m, 4H), 1.91 – 1.85 (m, 2H), 1.77 – 1.68 (m, 2H).

### O. *N*-(4-(5-(hydroxymethyl)-1*H*-imidazol-2-yl)butyl)acetamide trifluoroacetic acid 29

To a solution of (2-(4-aminobutyl)-1*H*-imidazol-5-yl)methanol **28** (50 mg, 0.3 mmol) in DCM/MeOH (1 ml/1 ml) was added acetic anhydride (6.1 mg, 0.06 mmol), and the mixture was stirred at room temperature overnight. The solvent was removed under reduced pressure, and the residue was purified by reverse phase chromatography (Biotage, C18 column, eluting with MeCN in water containing 0.1% TFA) to give the title compound (25 mg, 40%). LCMS-C: m/z 212.1 [M + H]^+^. ^1^H NMR (400 MHz, DMSO-d_6_) δ 14.2 (br s, 2H), 7.85 (t, J = 5.7 Hz, 1H), 7.38 (s, 1H), 5.63 (br s, 1H), 4.47 (s, 2H), 3.06 – 3.00 (m, 2H), 2.88 (t, J = 7.6 Hz, 2H), 1.78 (s, 3H), 1.72 – 1.64 (m, 2H), 1.41 – 1.33 (m, 2H).

### P. *N*-(4-(5-(hydroxymethyl)-1*H*-imidazol-2-yl)butyl)benzamide 30

To a solution of (2-(4-aminobutyl)-1*H*-imidazol-5-yl)methanol **28** (40 mg, 0.24 mmol) and benzoyl chloride (15 mg, 0.11 mmol) in DCM/MeOH (1 ml/1 ml) was added Et_3_N (72 mg, 0.72 mmol), and the mixture was stirred at room temperature overnight. The mixture was concentrated under reduced pressure, and the residue was purified by prep-TLC (DCM/MeOH = 10:1) to give the title compound (5 mg, 8%). LCMS-C: m/z 274.0 [M + H]^+^. ^1^H NMR (400 MHz, MeOD) δ 7.85 – 7.79 (m, 2H), 7.53 (t, J = 7.3 Hz, 1H), 7.47 – 7.44 (m, 2H), 7.24 (s, 1H), 4.59 (s, 2H), 3.43 (t, J = 6.9 Hz, 2H), 2.99 (t, J = 7.7 Hz, 2H), 1.88 – 1.79 (m, 2H), 1.72 – 1.64 (m, 2H).

### Q. *N*-(4-(5-(hydroxymethyl)-1*H*-imidazol-2-yl)butyl)-2-phenylacetamide trifluoroacetic acid 31

To a solution of (2-(4-aminobutyl)-1*H*-imidazol-5-yl)methanol **28** (30 mg, 0.18 mmol) in DCM (5 ml) and MeOH (1 ml) were added Et_3_N (75 *μ*L, 0.54 mmol) and phenylacetyl chloride (10 *μ*L, 0.07 mmol), and the mixture was stirred at room temperature overnight. The solvent was removed under reduced pressure, and the residue was diluted with water and washed with EtOAc. The aqueous layer was concentrated under reduced pressure, and the residue was purified by reverse phase chromatography (Biotage, C18 column, eluting with MeCN in water containing 0.1% TFA) to give the title compound (5 mg, 10%) as a yellow oil. LCMS-C: m/z 288.2 [M + H]^+^. ^1^H NMR (400 MHz, MeOD) δ 7.28 – 7.22 (m, 6H), 4.60 (s, 2H), 3.49 (s, 2H), 3.19 (t, J = 7.6 Hz, 2H), 2.93 (t, J = 7.6 Hz, 2H), 1.77 – 1.70 (m, 2H), 1.57 – 1.50 (m, 2H).

### R. Methyl (4-(5-(hydroxymethyl)-1*H*-imidazol-2-yl)butyl)carbamate trifluoroacetic acid 32

To a solution of (2-(4-aminobutyl)-1*H*-imidazol-5-yl)methanol **28** (50 mg, 0.3 mmol) in DCM (3 ml) and MeOH (1 ml) were added Et_3_N (0.13 ml, 0.9 mmol) and methyl chloroformate (23 *μ*l, 0.3 mmol), and the mixture was stirred at room temperature for 2 days. Water was added, and the mixture was washed with DCM. The aqueous layer was concentrated under reduced pressure, and the residue was purified by reverse phase chromatography (Biotage, C18 column, eluting with MeCN in water containing 0.1% TFA) to give the title compound (5 mg, 7%) as pale yellow oil. LCMS-C: m/z 228.2 [M + H]^+^. ^1^H NMR (400 MHz, DMSO-*d_6_*) δ 14.1 (br s, 2H), 7.39 (s, 1H), 7.10 (t, J = 5.4 Hz, 1H), 5.59 (br s, 1H), 4.46 (s, 2H), 3.49 (s, 3H), 3.01 – 2.95 (m, 2H), 2.87 (t, J = 7.6 Hz, 2H), 1.75 – 1.59 (m, 2H), 1.41 – 1.32 (m, 2H).

### S. Benzyl (4-(5-(hydroxymethyl)-1*H*-imidazol-2-yl)butyl)carbamate trifluoroacetic acid 35

#### 1. Step 1: Benzyl (4-cyanobutyl)carbamate 33

A mixture of 5-azidopentanenitrile **25** (3.8 g, 31 mmol) and 10% Pd/C (400 mg) in MeOH (40 ml) was stirred at room temperature under an atmosphere of hydrogen overnight. The mixture was filtered, and the filtrate was concentrated under reduced pressure. The residue was dissolved in DCM (40 ml), Et_3_N (6.2 g, 61 mmol) and Cbz-Cl (6.3 g, 37 mmol) were added, and the mixture was stirred at room temperature overnight. The mixture was concentrated under reduced pressure, and the residue was purified by silica gel chromatography (DCM/MeOH = 10:1) to give the title compound (2.6 g, 37%) as a colorless oil. LCMS-C: m/z 233.1 [M + H]^+^.

#### 2. Step 2: Benzyl (5-amino-5-iminopentyl)carbamate 34

To a solution of benzyl (4-cyanobutyl)carbamate **33** (460 mg, 1.98 mmol) in EtOH (5 ml) was added AcCl (1.2 g, 16 mmol) dropwise, and the mixture was heated at 30 °C overnight. The solvent was removed under reduced pressure, and the residue was taken up in EtOH and concentrated again under reduced pressure. The residue was dissolved in a 2 M NH_3_/MeOH solution (2 ml), and the mixture was stirred at room temperature overnight. The solvent was removed under reduced pressure, and the residue was purified by silica gel chromatography (DCM/MeOH = 1:0 to 7:1) to give the title compound (180 mg, 37%). LCMS-C: *m/z* 250.2 [M + H]^+^.

#### 3. Step 3: Benzyl (4-(5-(hydroxymethyl)-1*H*-imidazol-2-yl)butyl)carbamate trifluoroacetic acid 35

A mixture of benzyl (5-amino-5-iminopentyl)carbamate **34** (400 mg, 1.70 mmol) and 1,3-dihydroxypropan-2-one (153 mg, 1.70 mmol) in concentrated aqueous NH_4_OH (4 ml) was heated at 70 °C in a sealed tube for 4 h. The mixture was filtered, and the filtrate was concentrated under reduced pressure. The residue was purified by reverse phase chromatography (Biotage, C18 column, eluting with MeCN in water containing 0.1% TFA) to give the title compound (100 mg, 20%). LCMS-C: *M /z* 304.1 [M + H]^+^. ^1^H NMR (400 MHz, MeOD) δ 7.35 – 7.26 (m, 6H), 5.05 (s, 2H), 4.59 (s, 2H), 3.15 (t, *J *=* *6.9 Hz, 2H), 2.95 (t, *J *=* *7.7 Hz, 2H), 1.82 – 1.74 (m, 2H), 1.57 – 1.50 (m, 2H).

### T. Methyl benzyl(4-(5-(hydroxymethyl)-1*H*-imidazol-2-yl)butyl)carbamate trifluoroacetic acid 37

#### 1. Step 1: (2-(4-(Benzylamino)butyl)-1*H*-imidazol-5-yl)methanol 36

To a solution of (2-(4-aminobutyl)-1*H*-imidazol-5-yl)methanol **28** (30 mg, 0.18 mmol), benzaldehyde (19 mg, 0.18 mmol), and AcOH (11 *μ*l, 0.18 mmol) in MeOH (3 ml) was added NaCNBH_3_ (45 mg, 0.72 mmol), and the mixture was stirred at room temperature overnight. The mixture was concentrated under reduced pressure to give the title compound (47 mg, 100%), which was used in the next step without further purification. LCMS-C: *m/z* 260.1 [M + H]^+^.

#### 2. Step 2: Methyl benzyl(4-(5-(hydroxymethyl)-1H-imidazol-2-yl)butyl)carbamate trifluoroacetic acid 37

To a solution of (2-(4-(benzylamino)butyl)-1*H*-imidazol-5-yl)methanol (47 mg, 0.18 mmol) in DCM (3 ml) at 0 °C were added Et_3_N (0.10 ml, 0.72 mmol) and methyl chloroformate (28 *μ*l, 0.36 mmol), and the mixture was stirred at room temperature overnight. The mixture was concentrated under reduced pressure, and the residue was purified by prep. HPLC to give the title compound (4.5 mg, 8%). LCMS-C: *m/z* 318.2 [M + H]^+^. ^1^H NMR (400 MHz, DMSO-*d_6_*) δ 14.0 – 13.8 (m, 2H), 7.40 (s, 1H), 7.35 – 7.21 (m, 5H), 5.58 (br s, 1H), 4.47 (s, 2H), 4.42 (s, 2H), 3.62 (s, 3H), 3.21 – 3.13 (m, 2H), 2.85 (t, *J *=* *7.2 Hz, 2H), 1.67 – 1.60 (m, 2H), 1.49 – 1.40 (m, 2H).

### U. Methyl (4-(4-(hydroxymethyl)-1*H*-imidazol-2-yl)butyl)((6-oxo-1,6-dihydropyridin-3-yl)methyl)carbamate bis trifluoroacetic acid 40

#### 1. Step 1: *tert*-butyl 4-(((4-(5-(hydroxymethyl)-1H-imidazol-2-yl)butyl)amino)methyl)piperidine-1-carboxylate 38

To a solution of (2-(4-aminobutyl)-1*H*-imidazol-5-yl)methanol **28** (50 mg, 0.30 mmol), *tert*-butyl 4-formylpiperidine-1-carboxylate (63 mg, 0.30 mmol), and AcOH (18 mg, 0.30 mmol) in MeOH (10 ml) was added NaCNBH_3_ (75 mg, 1.20 mmol), and the mixture was stirred at room temperature overnight. The mixture was concentrated under reduced pressure to give the title compound (110 mg, 100%), which was used in the next step without further purification. LCMS-C: m/z 367.1 [M + H]^+^.

#### 2. Step 2: *tert*-butyl 4-(((4-(5-(hydroxymethyl)-1H-imidazol-2-yl)butyl)(methoxycarbonyl)amino)methyl)piperidine-1-carboxylate 39

To a solution of *tert*-butyl 4-(((4-(5-(hydroxymethyl)-1*H*-imidazol-2-yl)butyl)amino)methyl)piperidine-1-carboxylate **38** (110 mg, 0.30 mmol) and Et_3_N (0.17 ml, 1.2 mmol) in DCM (5 ml) at 0 °C was added methyl chloroformate (56.7 mg, 0.6 mmol), and the mixture was allowed to warm to room temperature and stirred overnight. The mixture was concentrated under reduced pressure, and the residue was purified by prep-HPLC to give the title compound (16 mg, 12%). LCMS-C: m/z 425.1 [M + H]^+^.

#### 3. Step 3: Methyl (4-(4-(hydroxymethyl)-1H-imidazol-2-yl)butyl)(piperidin-4-ylmethyl)carbamate bis trifluoroacetic acid 40

To a solution of *tert*-butyl 4-(((4-(5-(hydroxymethyl)-1*H*-imidazol-2-yl)butyl)(methoxycarbonyl)amino)methyl)piperidine-1-carboxylate **39** (16 mg, 0.038 mmol) in DCM (3 ml) at 0 °C was added TFA (0.2 ml), and the mixture was allowed to warm to room temperature and stirred for 4 h. The mixture was concentrated under reduced pressure to give the title compound (5 mg, 42%). LCMS-C: m/z 335.2 [M + H]^+^. ^1^H NMR (400 MHz, MeOD) δ 7.30 (s, 1H), 4.61 (s, 2H), 3.67 (s, 3H), 3.43 – 3.36 (m, 2H), 3.30 (2H obscured by solvent peak), 3.21 (d, J = 7.3 Hz, 2H), 3.02 – 2.90 (m, 4H), 2.05 – 1.93 (m, 1H), 1.91 – 1.81 (m, 2H), 1.80 – 1.72 (m, 2H), 1.65 – 1.57 (m, 2H), 1.48 – 1.36 (m, 2H).

### V. Methyl (4-(4-(hydroxymethyl)-1*H*-imidazol-2-yl)butyl)((6-oxo-1,6-dihydropyridin-3-yl)methyl)carbamate trifluoroacetic acid 45

#### 1. Step 1: 2-(4-azidobutyl)-5-(((*tert*-butyldiphenylsilyl)oxy)methyl)-1H-imidazole 41

To a solution of (2-(4-azidobutyl)-1*H*-imidazol-5-yl)methanol **27** (500 mg, 2.56 mmol) in DCM (10 ml) at 0 °C was added Et_3_N (1.1 ml, 7.68 mmol) followed by TBDPSCl (1.35 ml, 5.13 mmol), and the mixture was allowed to warm to room temperature and stirred for 2 days. The mixture was diluted with water and extracted with DCM, and the organic layer was washed with brine, dried over Na_2_SO_4_, filtered, and concentrated under reduced pressure. The residue was purified by silica gel chromatography (Pet. Ether/EtOAc = 10:1 to 5:1 to 1:1) to give the title compound (420 mg, 38%). LCMS-C: *m/z* 434.2 [M + H]^+^. ^1^H NMR (400 MHz, MeOD) δ 7.68 – 7.66 (m, 4H), 7.46 – 7.36 (m, 6H), 6.62 (s, 1H), 4.64 (s, 2H), 2.70 (t, *J *=* *7.6 Hz, 2H), 1.80 – 1.72 (m, 2H), 1.63 – 1.55 (m, 2H), 1.36 – 1.21 (m, 2H), 1.03 (s, 9H).

#### 2. Step 2: 4-(5-(((*tert*-butyldiphenylsilyl)oxy)methyl)-1H-imidazol-2-yl)butan-1-amine 42

A mixture of 2-(4-azidobutyl)-5-(((*tert*-butyldiphenylsilyl)oxy)methyl)-1*H*-imidazole **41** (350 mg, 0.81 mmol) and 10% Pd/C (350 mg) in MeOH (5 ml) was stirred at room temperature under an atmosphere of hydrogen overnight. The mixture was filtered, and the filtrate was concentrated under reduced pressure. The residue was purified by silica gel chromatography (DCM/MeOH = 20:1 to 10:1 to 5:1) to give the title compound (310 mg, 94%) as a yellow oil. LCMS-C: *m/z* 408.3 [M + H]^+^. ^1^H NMR (400 MHz, CDCl_3_) δ 7.69 – 7.64 (m, 4H), 7.45 – 7.33 (m, 6H), 6.71 (s, 1H), 4.69 (s, 2H), 2.76 (t, *J *=* *6.7 Hz, 2H), 2.68 (t, *J *=* *7.4 Hz, 2H), 1.80 – 1.69 (m, 2H), 1.60 – 1.48 (m, 2H), 1.05 (s, 9H).

#### 3. Step 3: 5-(((4-(4-(((*tert*-butyldiphenylsilyl)oxy)methyl)-1H-imidazol-2-yl)butyl)amino)methyl)pyridin-2(1H)-one 43

To a solution of 4-(5-(((*tert*-butyldiphenylsilyl)oxy)methyl)-1*H*-imidazol-2-yl)butan-1-amine **42** (120 mg, 0.29 mmol) and 6-hydroxynicotinaldehyde (37 mg, 0.29 mmol) in MeOH (10 ml) was added AcOH (0.15 ml), and the mixture was stirred at room temperature overnight. NaCNBH_3_ (73 mg, 1.2 mmol) was added, and the mixture was heated at 40 °C for 5 h. The mixture was diluted with water and extracted with DCM, and the organic extracts were washed with brine, dried over Na_2_SO_4_, filtered, and concentrated under reduced pressure. The residue was purified by reverse phase chromatography (Biotage, C18 column, eluting with MeCN in water) to give the title compound (150 mg, 100%). LCMS-C: *m/z* 515.1 [M + H]^+^.

#### 4. Step 4: Methyl (4-(4-(hydroxymethyl)-1H-imidazol-2-yl)butyl)((6-oxo-1,6-dihydropyridin-3-yl)methyl)carbamate trifluoroacetic acid 45

To a solution of 5-(((4-(4-(((*tert*-butyldiphenylsilyl)oxy)methyl)-1*H*-imidazol-2-yl)butyl)amino)methyl)pyridin-2(1H)-one **43** (50 mg, 0.13 mmol) in DCM (4 ml) were added Et_3_N (56 *μ*l, 0.4 mmol) and methyl chloroformate (8 *μ*l, 0.1 mmol), and the mixture was stirred at room temperature overnight. LCMS analysis revealed that only TBDPS deprotection had occurred. The mixture was extracted with ether and the organic extract was concentrated under reduced pressure. The residue was dissolved in DCM/MeOH (4 ml/0.5 ml) and treated with Et_3_N (100 *μ*l, 0.52 mmol) and methyl chloroformate (10 *μ*l, 0.13 mmol), and the mixture was stirred at room temperature overnight. MeOH (5 ml) and a 10% aqueous NaOH solution (0.5 ml) were added, and stirring was continued at room temperature overnight. The solvent was removed under reduced pressure, and the residue was purified by reverse phase chromatography (Biotage, C18 column, eluting with MeCN in water containing 0.1% TFA) to give the title compound (5 mg, 5%). LCMS-C: *m/z* 335.1 [M + H]^+^. ^1^H NMR (400 MHz, MeOD) δ 7.60 – 7.51 (m, 1H), 7.37 – 7.28 (m, 1H), 6.85 (s, 1H), 6.51 (d, *J *=* *9.3 Hz, 1H), 4.50 (s, 2H), 4.24 (s, 2H), 3.71 (s, 3H), 3.24 (t, *J *=* *7.4 Hz, 2H), 2.67 (t, *J *=* *7.2 Hz, 2H), 1.69 – 1.61 (m, 2H), 1.54 – 1.45 (m, 2H).

### W. Methyl (4-(4-(hydroxymethyl)-1*H*-imidazol-2-yl)butyl)((2-oxo-1,2-dihydropyridin-4-yl)methyl)carbamate hydrochloride 47

#### 1. Step 1: 4-(((4-(4-(((*tert*-butyldiphenylsilyl)oxy)methyl)-1H-imidazol-2-yl)butyl)amino)methyl)pyridin-2(1H)-one 44

A solution of 4-(5-(((*tert*-butyldiphenylsilyl)oxy)methyl)-1*H*-imidazol-2-yl)butan-1-amine **42** (0.43 g, 1.1 mmol) and 2-hydroxyisonicotinaldehyde (0.20 g, 1.6 mmol) in MeOH (20 ml) was stirred at room temperature overnight. NaCNBH_3_ (0.13 g, 2.12 mmol) and AcOH (2 drops) were added, and stirring was continued at room temperature for 4 h. The mixture was diluted with water and extracted with DCM. The combined organic extracts were washed with brine, dried over Na_2_SO_4_, filtered, and concentrated under reduced pressure to give the crude title compound (0.6 g), which was used directly in the next step. LCMS-C: *m/z* 515.0 [M + H]^+^.

#### 2. Step 2: Methyl (4-(4-(((*tert*-butyldiphenylsilyl)oxy)methyl)-1H-imidazol-2-yl)butyl)((2-oxo-1,2-dihydropyridin-4-yl)methyl)carbamate 46

To a solution of 4-(((4-(4-(((*tert*-butyldiphenylsilyl)oxy)methyl)-1H-imidazol-2-yl)butyl)amino)methyl)pyridin-2(1H)-one **44** (0.6 g, assumed 1.1 mmol) in DCM (10 ml) was added K_2_CO_3_ (0.29 g, 2.1 mmol) followed by a solution of methyl chloroformate (150 mg, 1.6 mmol) in DCM (2 ml) dropwise, and the mixture was stirred at room temperature for 2 h. The mixture was diluted with water and extracted with DCM, and the combined organic extracts were washed with brine, dried over Na_2_SO_4_, filtered, and concentrated under reduced pressure. The residue was purified by prep-HPLC to give the title compound (45.2 mg, 7%). LCMS-C: *m/z* 573.0 [M + H]^+^.

#### 3. Step 3: Methyl (4-(4-(hydroxymethyl)-1H-imidazol-2-yl)butyl)((2-oxo-1,2-dihydropyridin-4-yl)methyl)carbamate hydrochloride 47

A mixture of methyl (4-(4-(((*tert*-butyldiphenylsilyl)oxy)methyl)-1*H*-imidazol-2-yl)butyl)((2-oxo-1,2-dihydropyridin-4-yl)methyl)carbamate **46** (45 mg, 0.08 mmol) and a 4 M HCl in 1,4-dioxane solution (5 ml) was stirred at room temperature for 2 h. The solvent was removed under reduced pressure, and the residue was purified by prep-HPLC to give the title compound (3.2 mg, 12%). LCMS-C: *m/z* 335.2 [M + H]^+^. ^1^H NMR (400 MHz, MeOD) δ 7.40 – 7.31 (m, 2H), 6.46– 6.31 (m, 2H), 4.61 (s, 2H), 4.39 (s, 2H), 3.70 (s, 3H), 3.55 – 3.51 (m, 2H), 2.96 – 2.91 (m, 2H), 1.76 – 1.60 (m, 4H).

### X. N-(4-(5-(hydroxymethyl)-1*H*-imidazol-2-yl)butyl)acrylamide 49

#### 1. Step 1: N-(4-(5-(((*tert*-butyldiphenylsilyl)oxy)methyl)-1H-imidazol-2-yl)butyl)acrylamide 48

To a solution of 4-(5-(((*tert*-butyldiphenylsilyl)oxy)methyl)-1*H*-imidazol-2-yl)butan-1-amine **42** (400 mg, 0.98 mmol) in DCM (10 ml) at 0 °C was added a solution of NaOH (43 mg, 1.1 mmol) in water (1.0 ml) followed by a solution of acryloyl chloride (89 mg, 0.98 mmol) in DCM (2.0 ml) dropwise. The mixture was stirred at 0 °C for 2 h and then partitioned between DCM and water. The layers were separated, and the organic layer was dried over Na_2_SO_4_, filtered, and concentrated under reduced pressure to give the title compound (420 mg, 93%), which was used directly in the next step.

#### 2. Step 2: N-(4-(5-(hydroxymethyl)-1H-imidazol-2-yl)butyl)acrylamide 49

To a solution of N-(4-(5-(((*tert*-butyldiphenylsilyl)oxy)methyl)-1*H*-imidazol-2-yl)butyl)acrylamide **48** (260 mg, 0.56 mmol) in THF (10 ml) at 0 °C was added TBAF (1 M solution in THF, 1.4 ml, 1.4 mmol), and the mixture was allowed to warm to room temperature and stirred for 2 h. The solvent was removed under reduced pressure, and the residue was purified by reverse phase chromatography (Biotage, C18 column, eluting with MeCN in water) to give the title compound (5.0 mg, 4%). LCMS-C: *m/z* 224.2 [M + H]^+^. ^1^H NMR (400 MHz, D_2_O) δ 7.23 (s, 1H), 6.27 – 6.11 (m, 2H), 5.72 (dd, *J *=* *9.9, 1.6 Hz, 1H), 4.62 (s, 2H), 3.27 (t, *J *=* *6.8 Hz, 2H), 2.97 (t, *J *=* *7.5 Hz, 2H), 1.82 – 1.74 (m, 2H), 1.60 – 1.52 (m, 2H).

### Y. Peptide-imidazole hybrids

#### 1. 2-(2-acetamidopropanamido)-N-(1-amino-3-methyl-1-oxobutan-2-yl)-5-(4-(hydroxymethyl)-1H-imidazol-2-yl)pentanamide 55

##### a. Step 1: 4-(((*tert*-butyldimethylsilyl)oxy)methyl)-1-((2-(trimethylsilyl)ethoxy)methyl)-1H-imidazole/5-(((*tert*-butyldimethylsilyl)oxy)methyl)-1-((2-(trimethylsilyl)ethoxy)methyl)-1H-imidazole **50**

Lithium hexamethyldisilazide (1 M in toluene) (2.6 ml, 2.6 mmol) was added to a stirring solution of 4-(((*tert*-butyldimethylsilyl)oxy)methyl)-1*H*-imidazole **18** (0.370 g, 1.74 mmol) in THF (8.7 ml) under N_2_ at 0 °C. The reaction mixture was stirred for 15 min before SEMCl (0.46 ml, 2.6 mmol) was added. The resulting solution was stirred for 4 h. A saturated solution of NaHCO_3_ (10 ml) was added, and the mixture was extracted with EtOAc (3 × 10 ml). The combined organic layers were washed with water (10 ml) and brine (10 ml), dried (Na_2_SO_4_), and concentrated *in vacuo* to give an orange oil. The material was purified by column chromatography (24 g SiO_2_ cartridge, 2%–10% methanol in DCM) to give the title compound as a colorless oil in an ∼2:1 mixture of regioisomers (0.464 g, 77%). LCMS-B rt 3.29 min (@ 214 nm); (m/z) 343 [M + H]^+^. Major regioisomer: ^1^H NMR (400 MHz, Chloroform-d) δ 7.53 (t, J = 0.8 Hz, 1H), 6.94 (d, J = 0.8 Hz, 1H), 5.35 (s, 2H), 4.71 (s, 2H), 3.54–3.44 (m, 2H), 0.95–0.83 (m, J = 0.6 Hz, 11H), 0.06 (d, J = 0.6 Hz, 6H), -0.02 (s, 9H). Minor regioisomer: ^1^H NMR (400 MHz, Chloroform-d) δ 7.52 – 7.50 (m, 1H), 6.96 – 6.94 (m, 1H), 5.22 (d, J = 0.6 Hz, 2H), 4.71 (s, 2H), 3.52 – 3.42 (m, 2H), 0.95–0.83 (m, 11H), 0.10 (d, J = 0.6 Hz, 6H), -0.02 (s, 9H).

##### b. Step 2: 4-(((*tert*-butyldimethylsilyl)oxy)methyl)-2-iodo-1-((2-(trimethylsilyl)ethoxy)methyl)-1H-imidazole/5-(((*tert*-butyldimethylsilyl)oxy)methyl)-2-iodo-1-((2-(trimethylsilyl)ethoxy)methyl)-1H-imidazole **51**

*n*-BuLi (1.54 M in hexanes, 0.97 ml, 1.5 mmol) was added dropwise over 10 min to a stirring solution of 4-(((*tert*-Butyldimethylsilyl)oxy)methyl)-1-((2-(trimethylsilyl)ethoxy)methyl)-1*H*-imidazole/5-(((*tert*-butyldimethylsilyl)oxy)methyl)-1-((2-(trimethylsilyl)ethoxy)methyl)-1*H*-imidazole **50** (0.343 g, 1.00 mmol) in THF (10 ml) at −78 °C under N_2_. The resulting yellow solution was stirred at −78 °C for 1 h. A solution of iodine (0.381 g, 1.5 mmol) in THF (3 ml) was added dropwise until the orange/brown color persisted. The solution was stirred for 30 min. A saturated solution of sodium thiosulfate (10 ml) was added, and the aqueous was extracted with EtOAc (3 × 10 ml). The combined organic extracts were washed with water (10 ml) and brine (10 ml), dried (Na_2_SO_4_), and concentrated *in vacuo*. The material was purified by column chromatography (24 g SiO_2_ cartridge, 5%–100% EtOAc in petroleum benzine 40–60 °C) to give the title compound as a colorless oil in an ∼2:1 mixture of regioisomers (331 mg, 71%). LCMS-B: rt 3.97 min; m/z 468.8 [M + H]^+^. Major regioisomer: ^1^H NMR (400 MHz, Chloroform-d) δ 6.98 (s, 1H), 5.35 (s, 2H), 4.72 (d, J = 0.7 Hz, 2H), 3.58 – 3.50 (m, 2H), 0.95–0.87 (m, 11H), 0.07 (s, 6H), -0.00 (s, 9H). Minor regioisomer: ^1^H NMR (400 MHz, Chloroform-*d*) δ 7.06 (s, 1H), 5.19 (s, 2H), 4.69 (d, J = 0.7 Hz, 2H), 3.58 – 3.50 (m, 2H), 0.95–0.87 (m, 11H), 0.09 (s, 6H), −0.00 (s, 9H).

##### c. Step 3: 2-(2-acetamidopropanamido)-*N*-(1-amino-3-methyl-1-oxobutan-2-yl)-5-(4-(((tert-butyldimethylsilyl)oxy)methyl)-1-((2-(trimethylsilyl)ethoxy)methyl)-1H-imidazol-2-yl)pent-4-ynamide/2-(2-acetamidopropanamido)-*N*-(1-amino-3-methyl-1-oxobutan-2-yl)-5-(5-(((*tert*-butyldimethylsilyl)oxy)methyl)-1-((2-(trimethylsilyl)ethoxy)methyl)-1*H*-imidazol-2-yl)pent-4-ynamide **53**

A suspension of 4-(((*tert*-Butyldimethylsilyl)oxy)methyl)-2-iodo-1-((2-(trimethylsilyl)ethoxy)methyl)-1*H*-imidazole/5-(((*tert*-butyldimethylsilyl)oxy)methyl)-2-iodo-1-((2-(trimethylsilyl)ethoxy)methyl)-1*H*-imidazole **51** (50.0 mg, 0.107 mmol), 2-(2-acetamidopropanamido)-*N*-(1-amino-3-methyl-1-oxobutan-2-yl)pent-4-ynamide **52** (38 mg, 0.117 mmol) and CuI (1.2 mg, 0.006 mmol) in Et_3_N (0.5 ml) and DMF (1 ml) was bubbled with N_2_ and sonicated. PdCl_2_(PPh_3_)_2_ (2.2 mg, 0.003 mmol) was added, and the suspension was stirred at room temperature until TLC indicated complete consumption of alkyne. A saturated solution of ammonium chloride (10 ml) was added, and the resulting suspension was extracted with EtOAc (3 × 30 ml). The organic extracts were combined, washed with 50% brine (3 × 20 ml), dried (Na_2_SO_4_), and concentrated *in vacuo*. The mixture was purified by column chromatography (12 g SiO_2_ cartridge, 0%–15% MeOH in DCM) to give the desired compound as an ∼2:1 mixture of regioisomers (5.7 mg, 8%). LCMS-B rt 3.51 min; m/z mass ion not detected. Major regioisomer: ^1^H NMR (400 MHz, Methanol-*d_4_*) δ 6.90 (s, 1H), 5.50 (s, 2H), 4.77 (s, 2H), 4.71 – 4.65 (m, 1H), 4.34 – 4.26 (m, 1H), 4.24 – 4.18 (m, 1H), 3.62 – 3.55 (m, 2H), 3.15 – 3.05 (m, 1H), 3.02 – 2.89 (m, 1H), 2.25 – 2.06 (m, 1H), 2.00 – 1.89 (m, 3H), 1.37 – 1.32 (m, 3H), 1.00 – 0.88 (m, 17H), 0.15 – 0.05 (m, 6H), 0.05 – -0.06 (m, 9H). Minor regioisomer: ^1^H NMR (400 MHz, Methanol-*d_4_*) δ 7.15 (s, 1H), 5.38 (d, J = 2.6 Hz, 2H), 4.60 (s, 2H), 4.71 – 4.65 (m, 1H), 4.34 – 4.26 (m, 1H), 4.24 – 4.18 (m, 1H), 3.62 – 3.55 (m, 2H), 3.15 – 3.05 (m, 1H), 3.02 – 2.89 (m, 1H), 2.25 – 2.06 (m, 1H), 2.00 – 1.89 (m, 3H), 1.37 – 1.27 (m, 5H), 1.00–0.88 (m, 15H), 0.15 – 0.05 (m, 6H), 0.05 – -0.06 (m, 9H).

##### d. Step 4: 2-(2-acetamidopropanamido)-*N*-(1-amino-3-methyl-1-oxobutan-2-yl)-5-(4-(((*tert*-butyldimethylsilyl)oxy)methyl)-1-((2-(trimethylsilyl)ethoxy)methyl)-1H-imidazol-2-yl)pentanamide/2-(2-acetamidopropanamido)-*N*-(1-amino-3-methyl-1-oxobutan-2-yl)-5-(5-(((*tert*-butyldimethylsilyl)oxy)methyl)-1-((2-(trimethylsilyl)ethoxy)methyl)-1H-imidazol-2-yl)pentanamide **54**

A solution of 2-(2-acetamidopropanamido)-*N*-(1-amino-3-methyl-1-oxobutan-2-yl)-5-(4-(((*tert*-butyldimethylsilyl)oxy)methyl)-1-((2-(trimethylsilyl)ethoxy)methyl)-1*H*-imidazol-2-yl)pent-4-ynamide/2-(2-acetamidopropanamido)-*N*-(1-amino-3-methyl-1-oxobutan-2-yl)-5-(5-(((*tert*-butyldimethylsilyl)oxy)methyl)-1-((2-(trimethylsilyl)ethoxy)methyl)-1*H*-imidazol-2-yl)pent-4-ynamide **53** (5.2 mg, 0.008 mmol) in EtOH (1.0 ml) was evacuated and back filled with N_2_ three times. Pd/C (53% water wet, 3.2 mg) was added, and the flask was evacuated and back-filled with N_2_ three times and then evacuated and back-filled with H_2_ from a balloon. The reaction mixture was stirred at room temperature overnight and then evacuated and back filled with N_2_ three times. The suspension was diluted with EtOAc (10 ml) and filtered through a pad of Celite and washed with EtOAc (10 ml). The solution was concentrated *in vacuo* to give the desired product (4.3 mg, 82%) as a mixture of regioisomers. LCMS-B rt 3.47 min (no absorbance at 254/214 nm); m/z 669 [M + H]^+^. Major regioisomer: ^1^H NMR (400 MHz, Methanol-*d_4_*) δ 6.80 (s, 1H), 5.38 (s, 2H), 5.37 – 5.33 (m, 1H), 4.74 (s, 2H), 4.35 – 4.25 (m, 1H), 4.21 – 4.15 (m, 1H), 3.61 – 3.56 (m, 2H), 2.89–2.69 (m, 2H), 2.19 (t, J = 7.6 Hz, 2H), 2.08 – 2.00 (m, 1H), 1.98 – 1.94 (m, 3H), 1.85 (m, 2H), 1.81 – 1.71 (m, 1H), 1.66 – 1.56 (m, 1H), 1.39 – 1.27 (m, 3H obscured), 0.99 – 0.84 (m, 15H), 0.08 (dd, J = 2.8, 1.0 Hz, 6H), -0.00 (s, 9H). Minor regioisomer: ^1^H NMR (400 MHz, Methanol-*d_4_*) δ 7.00 (s, 1H), 5.38 – 5.33 (m, 1H), 5.28 (s, 2H), 4.61 (s, 2H), 4.35 – 4.25 (m, 1H), 4.21 – 4.15 (m, 1H), 3.61 – 3.56 (m, 2H), 2.89–2.69 (m, 2H), 2.19 (t, J = 7.6 Hz, 2H), 2.08 – 2.00 (m, 1H), 1.98 – 1.94 (m, 3H), 1.85 (m, 2H), 1.81 – 1.71 (m, 1H), 1.66 – 1.56 (m, 1H), 1.39 – 1.27 (m, 3H obscured), 0.99 – 0.84 (m, 15H), 0.08 (dd, J = 2.8, 1.0 Hz, 6H), -0.00 (s, 9H).

##### e. Step 5: 2-(2-acetamidopropanamido)-*N*-(1-amino-3-methyl-1-oxobutan-2-yl)-5-(4-(hydroxymethyl)-1H-imidazol-2-yl)pentanamide **55**

TFA (0.5 ml) was added to a solution of 2-(2-Acetamidopropanamido)-*N*-(1-amino-3-methyl-1-oxobutan-2-yl)-5-(4-(((*tert*-butyldimethylsilyl)oxy)methyl)-1-((2-(trimethylsilyl)ethoxy)methyl)-1*H*-imidazol-2-yl)pentanamide/2-(2-acetamidopropanamido)-*N*-(1-amino-3-methyl-1-oxobutan-2-yl)-5-(5-(((*tert*-butyldimethylsilyl)oxy)methyl)-1-((2-(trimethylsilyl)ethoxy)methyl)-1*H*-imidazol-2-yl)pentanamide **54** (4.3 mg, 0.006 mmol) in DCM (0.5 ml) at room temperature. The solution was stirred overnight and then concentrated *in vacuo*. The residue was diluted in minimum MeOH and loaded onto an SCX cartridge (0.5 g, Silicycle), washed with MeOH (10 ml), and then eluted with 7 M NH_3_ in MeOH (3 × 5 ml). The residue was concentrated *in vacuo* to give the desired compound (1.1 mg, 40%) as a colorless oil. LCMS-B rt 0.54 min (no absorbance at 214/254 nm, rt based on +ve TIC chromatogram), m/z 425.0 [M + H]^+^. ^1^H NMR (400 MHz, Methanol-*d_4_*) δ 7.07 – 6.94 (m, 1H), 4.57 – 4.49 (m, 2H), 4.50 – 4.10 (m, 3H), 2.78 (m, 2H), 2.23 – 2.10 (m, 1H), 1.98 (dd, J = 7.8, 2.0 Hz, 3H), 1.92 – 1.64 (m, 4H), 1.40 – 1.31 (m, 3H), 1.01 – 0.80 (m, 6H). The product was isolated as a mixture of diastereomers.

### Z. 2-(2-acetamido-2-methylpropanamido)-*N*-(1-amino-3-methyl-1-oxobutan-2-yl)-5-(4-(hydroxymethyl)-1*H*-imidazol-2-yl)pentanamide 59

#### 1. Step 1: 2-(2-acetamido-2-methylpropanamido)-N-(1-amino-3-methyl-1-oxobutan-2-yl)-5-(4-(((*tert*-butyldimethylsilyl)oxy)methyl)-1-((2-(trimethylsilyl)ethoxy)methyl)-1H-imidazol-2-yl)pent-4-ynamide/2-(2-acetamido-2-methylpropanamido)-N-(1-amino-3-methyl-1-oxobutan-2-yl)-5-(5-(((*tert*-butyldimethylsilyl)oxy)methyl)-1-((2-(trimethylsilyl)ethoxy)methyl)-1H-imidazol-2-yl)pent-4-ynamide 57

A suspension of 4-(((*tert*-butyldimethylsilyl)oxy)methyl)-2-iodo-1-((2-(trimethylsilyl)ethoxy)methyl)-1*H*-imidazole/5-(((*tert*-butyldimethylsilyl)oxy)methyl)-2-iodo-1-((2-(trimethylsilyl)ethoxy)methyl)-1*H*-imidazole **51** (8.3 mg, 0.018 mmol) and 2-(2-acetamido-2-methylpropanamido)-*N*-(1-amino-3-methyl-1-oxobutan-2-yl)pent-4-ynamide **56** (6.0 mg, 0.018 mmol) in Et_3_N (0.5 ml) was degassed by three freeze (liq. N_2_)-pump-thaw cycles. CuI (0.4 mg, 1.8 *μ*mol) and PdCl_2_(PPh_3_)_2_ (1.2 mg, 1.8 *μ*mol) were added, and a freeze-pump-thaw cycle was repeated. The resulting suspension was stirred at room temperature for 4 days. Water (10 ml) was added, and the resulting suspension was extracted with EtOAc (3 × 30 ml). The organic extracts were combined, washed with water (30 ml) and brine (30 ml), dried (Na_2_SO_4_), and concentrated *in vacuo*. The mixture was purified by column chromatography (12 g SiO_2_ cartridge, 1%–15% MeOH in DCM) to give the title compound (1.2 mg, 10%). LCMS-A rt 6.17 min; m/z 679 [M + H]^+^, 677 [M-H]^−^.

#### 2. Step 2: 2-(2-acetamido-2-methylpropanamido)-N-(1-amino-3-methyl-1-oxobutan-2-yl)-5-(4-(((*tert*-butyldimethylsilyl)oxy)methyl)-1-((2-(trimethylsilyl)ethoxy)methyl)-1H-imidazol-2-yl)pentanamide/2-(2-acetamido-2-methylpropanamido)-N-(1-amino-3-methyl-1-oxobutan-2-yl)-5-(5-(((*tert*-butyldimethylsilyl)oxy)methyl)-1-((2-(trimethylsilyl)ethoxy)methyl)-1H-imidazol-2-yl)pentanamide 58

A solution of 2-(2-acetamido-2-methylpropanamido)-*N*-(1-amino-3-methyl-1-oxobutan-2-yl)-5-(4-(((*tert*-butyldimethylsilyl)oxy)methyl)-1-((2-(trimethylsilyl)ethoxy)methyl)-1*H*-imidazol-2-yl)pent-4-ynamide/2-(2-acetamido-2-methylpropanamido)-*N*-(1-amino-3-methyl-1-oxobutan-2-yl)-5-(5-(((*tert*-butyldimethylsilyl)oxy)methyl)-1-((2-(trimethylsilyl)ethoxy)methyl)-1*H*-imidazol-2-yl)pent-4-ynamide **57** (1.1 mg, 1.7 *μ*mol) in EtOH (1.0 ml) was evacuated and back filled with N_2_ three times. Pd/C (53% water wet, 1.2 mg) was added and the flask was evacuated and back-filled with N_2_ three times and then evacuated and back-filled with H_2_ from a balloon. The reaction mixture was stirred at room temperature overnight before being evacuated and back filled with N_2_ three times. The suspension was diluted with EtOAc (10 ml) and filtered through a pad of Celite that was then washed with EtOAc (10 ml). The solution was concentrated *in vacuo* to give the title compound that was used directly in the next step without further purification or characterization (assumed 100% conversion). LCMS-A rt 5.62 min (no absorbance at 214/254 nm, rt based on +ve TIC chromatogram), m/z 683 [M + H]^+^.

#### 3. Step 3: 2-(2-acetamido-2-methylpropanamido)-N-(1-amino-3-methyl-1-oxobutan-2-yl)-5-(4-(hydroxymethyl)-1*H*-imidazol-2-yl)pentanamide 59

TFA (0.5 ml) was added to a solution of 2-(2-acetamido-2-methylpropanamido)-*N*-(1-amino-3-methyl-1-oxobutan-2-yl)-5-(4-(((*tert*-butyldimethylsilyl)oxy)methyl)-1-((2-(trimethylsilyl)ethoxy)methyl)-1*H*-imidazol-2-yl)pentanamide/(S)-2-(2-acetamido-2-methylpropanamido)-*N*-(1-amino-3-methyl-1-oxobutan-2-yl)-5-(5-(((*tert*-butyldimethylsilyl)oxy)methyl)-1-((2-(trimethylsilyl)ethoxy)methyl)-1*H*-imidazol-2-yl)pentanamide **58** (1.76 *μ*mol) in DCM (0.5 ml) at room temperature. The solution was stirred overnight and then concentrated *in vacuo*. The residue was concentrated from MeOH *in vacuo* to give the title compound (0.6 mg, 68%) as a yellow oil. The compound was lypophilized from MeCN:H_2_O to give the title compound as an off-white oil (0.54 mg, 55% over 2 steps). LCMS-D rt 5.19 min, m/z 439.0 [M + H]^+^.

The tripeptide Ac-ARV-NH_2_ was purchased from Mimotopes (Victoria, Australia).

## SUPPLEMENTARY MATERIAL

See the supplementary material for X-ray crystallography collection and refinement statistics, ligand difference density maps, SPR sensorgrams, and a scheme for the synthesis of compounds **55** and **59**.
